# Phytochemical Profile and Antioxidant Potential of *Montanoa bipinnatifida* C. Koch Leaf Extract: Promising Bioactives for Pharmaceutical Applications

**DOI:** 10.3390/antiox15050598

**Published:** 2026-05-08

**Authors:** Verónica Edith Gallegos-Hernández, Ehekatzin García-Valdés, Maria Fernanda Vargas-Torrico, Gustavo G. Medina-Mendoza, Patricia Vergara-Aragón, Mónica Rosalía Jaime-Fonseca

**Affiliations:** 1 Centro de Investigación en Ciencia Aplicada y Tecnología Avanzada (CICATA), Unidad Legaria, Instituto Politécnico Nacional, Av. Legaria 694, Irrigación, Miguel Hidalgo, Mexico City 11500, Mexico; gavh940112@gmail.com (V.E.G.-H.); 09ehekatzin94@gmail.com (E.G.-V.); 2 Laboratorio de Procesos de Transformación y Tecnologías Emergentes de Alimentos, Facultad de Estudios Superiores Cuautitlán, Universidad Nacional Autónoma de México, Km 2.5 Carretera Cuautitlán–Teoloyucan, San Sebastián Xhala, Cuautitlán Izcalli 54714, Mexico; fervargast3@gmail.com; 3 Departamento de Biotecnología y Bioingeniería, Centro de Investigación y de Estudios Avanzados (CINVESTAV), Instituto Politécnico Nacional, Av. Instituto Politécnico Nacional 2508, San Pedro Zacatenco, Gustavo A. Madero, Mexico City 07360, Mexico; gustavo.medina@cinvestav.mx; 4 Departamento de Fisiología, Facultad de Medicina, Universidad Nacional Autónoma de México, Av. Universidad 3000, Coyoacán, Mexico City 04510, Mexico

**Keywords:** Asteraceae, metabolomic profiling, ESI-FT-ICR-MS, GC–MS

## Abstract

Plants long employed in traditional medicine remain promising yet underexplored sources of antioxidant compounds. *Montanoa bipinnatifida* C. Koch, native to southern Mexico, is one such species with a largely uncharacterized chemical profile. In this study, a hydroethanolic of *Montanoa bipinnatifida* C. Koch leaf extract (MB-LE) was prepared via maceration with 70% ethanol, yielding 18% (*w*/*w*), and subsequently analyzed using spectroscopic and metabolomic approaches. UV–Vis and FTIR spectra revealed a chemically diverse matrix enriched in phenolic and nitrogen-containing constituents. High-resolution ESI-FT-ICR-MS profiling enabled the tentative identification of structurally varied metabolites, including alkaloids, saponins, glycosides, coumarins, and small peptides, while GC–MS analysis further indicated the presence of terpenoids and lipid-related compounds. These findings highlight a previously unrecognized chemical complexity in these species. Functionally, MB-LE exhibited moderate antioxidant activity in the DPPH, ABTS, and FRAP assays (IC_50_ = 56.21 ± 0.67 µg/mL, 724.82 ± 27.49 µmol TE/g extract, and 614.90 ± 42.63 µmol TE/g extract, respectively), while the extract’s high phenolic content (333.31 ± 11.93 mg GAE/g extract) suggests a central role of these compounds in its observed activity. Overall, this work positions *M. bipinnatifida* as a novel source of bioactive metabolites and establishes a foundation for future studies focused on its structural elucidation and biological validation.

## 1. Introduction

*Montanoa bipinnatifida* C. Koch (Asteraceae) is a perennial shrub broadly distributed from Mexico to northern South America, with a notable presence in Mesoamerican ecosystems where the genus *Montanoa* reaches its highest diversity [1]. Comprising approximately 25 species, this genus has attracted attention due to its ecological importance and its capacity to biosynthesize structurally diverse secondary metabolites [2,3]. Previous phytochemical studies have shown that *Montanoa* species are particularly rich in terpenoids, including mono-, sesqui-, and triterpenes- as well as flavonoids, many of which have been linked to antioxidant and anti-inflammatory effects [4]. These metabolites are known to participate in the modulation of oxidative stress, a process closely associated with the onset and progression of chronic and degenerative diseases.

Within traditional medicine, *M. bipinnatifida* locally referred to as “Penumbra” has been employed for the treatment of wounds (especially those related to metabolic imbalances such as hyperglycemia) and in managing the effects of insect envenomation [5], indicating its potential role in regulating redox homeostasis and inflammatory responses. Nevertheless, despite its ethnopharmacological relevance, the chemical composition of this species and its antioxidant capacity have not yet been comprehensively examined [1,5,6].

Evidence from related species reinforces the pharmacological potential of the genus. For instance, *Montanoa quadrangularis* has been reported to contain monoterpenes such as myrcene, limonene, β-phellandrene, and sabinene, compounds that exhibit antimicrobial activity against bacterial strains of clinical relevance, including *Bacillus subtilis*, *Staphylococcus epidermidis*, and *Escherichia coli* [7]. Likewise, studies on *Montanoa frutescens*, *Montanoa grandiflora*, and *Montanoa tomentosa* have identified flavonoids such as quercetin and rutin, which are widely recognized for their antioxidant properties and their influence on neurochemical pathways [8]. In addition, *M. grandiflora* extract have demonstrated anti-inflammatory effects through the modulation of cytokines and oxidative mediators, including reductions in TNF-α, IL-1β, IL-6, nitric oxide (NO), and hydrogen peroxide (H_2_O_2_) [9].

Despite these advances, information on *M. bipinnatifida* remains scarce. In this context, the present study was designed to provide a detailed characterization of the phytochemical profile and antioxidant activity of a hydroethanolic leaf extract of this species. To this end, conventional phytochemical screening was integrated with spectroscopic techniques (UV–Vis and FTIR), chromatographic analysis (GC–MS), and high-resolution untargeted metabolomics using ESI-FT-ICR-MS, complemented by in vitro antioxidant assays.

## 2. Materials and Methods

### 2.1. Reagents

2,2-Diphenyl-1-picrylhydrazyl (DPPH), gallic acid, catechin, Folin–Ciocalteu reagent, sodium carbonate (Na_2_CO_3_), potassium persulfate (K_2_S_2_O_8_), acetonitrile, pyridine, N,O-bis(trimethylsilyl)trifluoroacetamide (BSTFA, 1% TMCS), and methanol (LC–MS grade) were obtained from Sigma-Aldrich (St. Louis, MO, USA). Trolox (Sigma Aldrich, St. Louis, Mo, USA) was used as the reference antioxidant standard. All solutions were prepared with double-distilled water.

### 2.2. Plant Material

Leaves of *Montanoa bipinnatifida* C. Koch were collected in September of 2022 from a nursery in Berriozábal, Chiapas, Mexico (16°47′ N, 93°16′ W; 820 m a.s.l.). The material was cleaned, disinfected with 1% (*v*/*v*) sodium hypochlorite for 5 min, rinsed thoroughly with distilled water, and air-dried at room temperature (25 ± 2 °C) for 15 days. The dried leaves were ground in a disk mill (Model 148–2, The Bauer Co., Worthington, PA, USA), sieved (300 µm), and stored at 4 °C until extraction.

### 2.3. Preparation of the Extract

Dried plant material (300 g) was subjected to maceration with 70% ethanol (1:10, *w*/*v*) at room temperature (22 ± 2 °C) in the absence of light for 15 days [10]. The extract was filtered using Whatman No. 1.5 filter paper (Whatman International, Ltd., Maidstone, UK) and concentrated under reduced pressure at 45 ± 2 °C using a rotary evaporator (RE-500, Yamato, Tokyo, Japan). Residual solvent was removed at room temperature (22 ± 2 °C), and the resulting dry extract (MB-LE) was stored in amber containers at 4 °C until further analysis.

### 2.4. Physicochemical and Phytochemical Characterization

#### 2.4.1. UV–Vis Spectroscopy

The MB-LE samples were preliminarily characterized using UV–Vis spectrophotometry (Genesys 10S, Thermo Scientific, Waltham, MA, USA) over a wavelength range of 200–800 nm. The analysis was performed to identify characteristic absorption bands associated with conjugated systems and chromophoric groups present in the extract, particularly those related to phenolic compounds, flavonoids, and other secondary metabolites.

Prior to measurement, the extract was diluted in an 80% methanol solution to ensure absorbance values within the linear range of the instrument. The solvent was used as a blank, and spectra were recorded at room temperature (22 ± 2 °C) under standard conditions.

#### 2.4.2. FTIR Analysis

Fourier transform infrared (FTIR) spectra of the MB-LE were recorded using an IRAffinity-1S spectrometer (Shimadzu, Kyoto, Japan) equipped with an attenuated total reflectance (ATR) accessory. Spectra were acquired over the wavenumber range of 4000–650 cm^−1^, with a spectral resolution of 2 cm^−1^ and 32 accumulated scans per sample.

Prior to analysis, samples were directly applied onto the ATR crystal without further preparation. Background spectra were collected under identical conditions and automatically subtracted from each sample spectrum. All measurements were performed at room temperature and in triplicate.

#### 2.4.3. ESI-FT-ICR-MS Analysis

Untargeted metabolomic profiling of the MB-LE was performed using a Solarix XR 7 T FT-ICR-MS system (Bruker Daltonics, Bremen, Germany) equipped with an electrospray ionization (ESI) source. Samples were prepared at a concentration of 1 mg/mL in a methanol/water mixture (1:1, *v*/*v*), filtered through a 0.22 µm PVDF membrane (Millipore, Burlington, MA, USA), and directly infused into the mass spectrometer at a flow rate of 120 µL/h.

Data acquisition was carried out in both positive and negative ionization modes (ESI+ and ESI−) over an *m*/*z* range of 150–3000 (16 scans/s), with a capillary voltage of 4500 V, end-plate offset of 500 V, 1M acquisition size, and ion accumulation time of 0.1 s, allowing for comprehensive detection of metabolites with diverse polarity and molecular weights. Nitrogen was used as a nebulizer/drying gas (1.0 bar, 2.0 L min^−1^ at 100 °C). Sodium trifluoroacetate was employed for external calibration.

Molecular formula assignment was performed using the MetaboScape 2022b software (Bruker Daltonics, Bremen, Germany; 2022b version), applying a mass accuracy threshold of ≤5 ppm [11,12]. Metabolite annotations were considered putative (level 3) according to the Metabolomics Standards Initiative (MSI), as they were based on accurate mass and database matching without MS/MS confirmation.

#### 2.4.4. GC–MS Analysis

Gas chromatography–mass spectrometry (GC–MS) analysis of the MB-LE was carried out using a CLARUS 580 gas chromatograph coupled to an SQ8S mass spectrometer (PerkinElmer, Shelton, CT, USA), equipped with an Elite-5 MS capillary column (30 m × 0.32 mm i.d., 0.25 µm film thickness).

The oven temperature program was set from 50 °C (for 3 min) to 200 °C (6 °C/min) and then to 260 °C (4 °C/min) with a controlled heating ramp. Helium was used as the carrier gas at a constant flow rate of 0.8 mL/min.

The mass spectrometer operated under electron ionization (EI) mode at 70 eV, and mass spectra were acquired over a 30–500 *m*/*z* range (10 scans/s) to ensure the detection of volatile and non-volatile compounds.

To analyze the volatile compounds, present in the MB-LE, 3 mg of the extract was diluted in 10 mL of pure methanol and filtered through a 0.22 µm PVDF hydrophilic membrane filter (Millipore, Burlington, MA, USA). Then, 3 µL of the diluted extract was directly injected into the equipment for the identification of volatile compounds. To determine non-volatile compounds, the solvent was first evaporated from 200 µL of the MB-LE at 50 °C for 30 s. Then derivatization was achieved by adding 160 µL of BSTFA + TMS reagent and 100 µL of pyridine, after which the mixture was incubated at 80 °C for 30 min. Following this incubation period, 3 µL of the formed derivative was injected into the equipment.

Compound identification was performed by comparing the obtained mass spectra with those in the NIST (National Institute of Standards and Technology) mass spectral library, considering similarity indices ≥ 80% as acceptable for tentative identification.

### 2.5. Antioxidant Activity

#### 2.5.1. DPPH Radical Scavenging Assay

Radical scavenging activity was evaluated using the DPPH method [13]. Briefly, different concentrations of the extract were mixed with a DPPH solution (to a final concentration of 0.2 mM) and incubated in the dark for 30 min. Absorbance was then measured at 515 nm. In this assay, a solution consisting of 200 µL of 80% methanol and 50 µL of DPPH was used as the control, while 250 µL of 80% methanol was used as the blank. The percentage of DPPH radical scavenging activity was calculated using Equation (1).

(1) $$\%{DPPH}_{degraded}=\left[1-\frac{Am-Ab}{Ac-Ab}\right]\times 100$$

where *Am* is the absorbance of the sample, *Ab* is the absorbance of the blank (250 µL of 80% MeOH), and *Ac* is the absorbance of the control (200 µL of 80% methanol with 50 µL of DPPH).

The IC_50_ value was determined by plotting the percentage of DPPH scavenging activity as a function of the extract concentration. Antioxidant capacity is expressed as µg of dry extract per mL (µg/mL), and the results are expressed as IC_50_ values (µg/mL).

#### 2.5.2. Analysis of Total Antioxidant Capacity Using the ABTS^•+^ Method

The ABTS^•+^ cation radical was generated by reacting 7.4 mM ABTS with 2.6 mM K_2_S_2_O_8_, then incubating the mixture for 16 h in the dark at room temperature [14]. The resulting ABTS^•+^ solution was then diluted with methanol to an absorbance of 0.70–1.2 at 734 nm, measured using a UV–Vis spectrophotometer (Thermo Scientific, Genesys 10S).

To assess antioxidant activity, aliquots of the extract were mixed with the ABTS^•+^ solution, and the decrease in absorbance at 734 nm was measured after a predetermined reaction time (10 min). The results are expressed as micromoles of Trolox equivalents (µmol TE) per gram of extract (µmol TE/g extract). Each trial was performed in triplicate, and values are reported as mean ± standard deviation.

#### 2.5.3. Analysis of Iron-Reducing Capacity Using the FRAP Method

The FRAP reagent was freshly prepared by mixing acetate buffer (pH 3.6), TPTZ solution (0.01 M in 0.04 M HCl), and FeCl_3_·6H_2_O solution (0.02 M) in a 10:1:1 (*v*/*v*/*v*) ratio [15]. A 20 µL aliquot of the extract was incubated with the FRAP reagent (180 µL) at room temperature for 30 min in the dark. After incubation, absorbance was measured at 593 nm using a UV–Vis spectrophotometer (Thermo Scientific, Genesys 10S).

Antioxidant activity is expressed as micromoles of Trolox equivalents (µmol TE) per gram of extract (µmol TE/g extract). All assays were performed in triplicate, and the results are reported as mean ± standard deviation.

### 2.6. Determination of Total Phenolic Compounds

The total phenolic content (TPC) was determined according to the method of Singleton and Rossi (1965) with slight modifications, using gallic acid as the calibration standard [16]. Briefly, 25 µL of each sample was mixed with 125 µL of distilled water, followed by the addition of 20 µL of Folin–Ciocalteu reagent, previously diluted 1:10 (*v*/*v*), and 30 µL of 20% (*w*/*w*) sodium carbonate solution. The reaction mixture was incubated in the dark for 30 min at room temperature. Absorbance was then measured at 760 nm using a Multiskan Go microplate spectrophotometer (Thermo Fisher Scientific, Vantaa, Finland).

A calibration curve was constructed using gallic acid, and the results are expressed as milligrams of gallic acid equivalents per gram of dry extract (mg GAE/g extract). All tests were performed in triplicate, and values are reported as mean ± standard deviation.

### 2.7. Statistical Analysis

All experiments were conducted using *n* = 3 independent biological replicates, each analyzed in triplicate. Results are expressed as mean ± standard deviation (SD). Data normality and homogeneity of variances were evaluated using the Shapiro–Wilk and Levene tests, respectively. Since the assumptions of normality and homogeneity of variances were met, statistical differences among groups were assessed via one-way analysis of variance (ANOVA), followed by the Holm–Sidak post hoc test for multiple comparisons. The significance level of *p* < 0.05 was considered statistically significant. All statistical analyses were performed using the SigmaPlot software (version 15.0; Systat Software Inc., San Jose, CA, USA).

## 3. Results

### 3.1. Yield Obtained via Maceration by Extraction

The maceration process yielded 36.4 g of MB-LE, corresponding to 18.18% of the initial dry weight of the plant material. Extraction efficiency is influenced by several factors, including solvent polarity, extraction time, temperature, and particle size of the plant matrix [17,18].

Maceration is a diffusion-driven extraction technique in which soluble compounds are gradually released from the plant matrix into the solvent due to a concentration gradient, without the application of external mechanical or thermal energy. Although this method is relatively simple and suitable for preserving thermolabile compounds, it generally requires longer extraction times compared to more intensive extraction techniques. A hydroalcoholic ethanol/water mixture (7:3, *v*/*v*) was employed for the preparation of MB-LE, as this solvent system is widely recognized for its effectiveness in extracting both polar and semipolar metabolites. Previous studies have shown that this ratio enhances the solubility of a wide range of phytochemicals while reducing the toxicity associated with purely organic solvents [19,20].

The effectiveness of this solvent system can be attributed to its intermediate polarity, which facilitates the extraction of a broad spectrum of bioactive compounds. In addition, the presence of water promotes matrix swelling and improves solvent penetration, whereas ethanol reduces surface tension and enhances mass transfer. However, excessively high ethanol proportions may negatively affect extraction yield by limiting the solubility of highly polar compounds or promoting the degradation of sensitive metabolites [18].

### 3.2. UV–Vis Spectrophotometric Analysis of MB-LE

**Figure 1:**
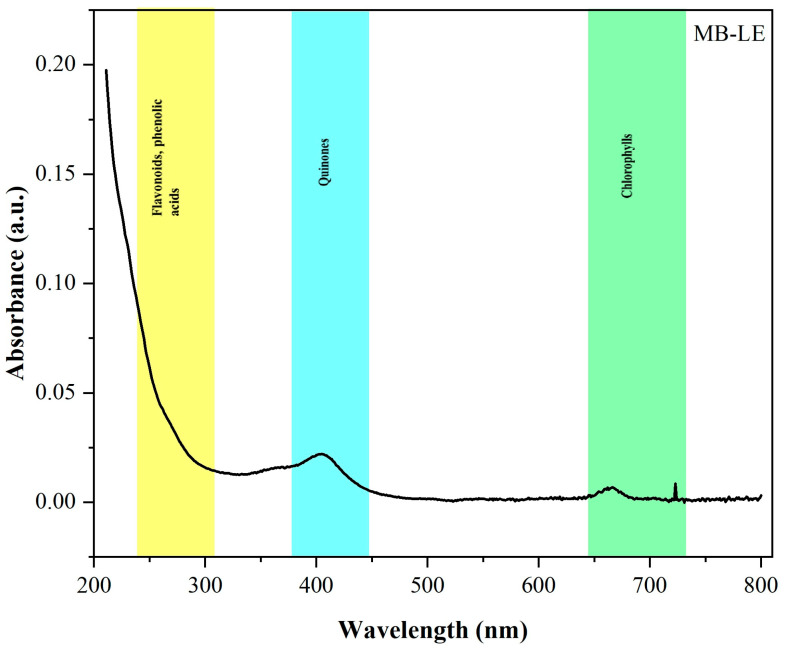
UV–Vis absorption spectrum of MB-LE, showing maxima at 250, 270, 300, 400, and 657 nm, indicative of conjugated aromatic and pigment-related compounds.

The UV–Vis spectrophotometric analysis of MB-LE revealed the presence of five maximum absorption peaks at 250, 270, 300, 400, and 657 nm (Figure 1). Absorption bands in the near-UV region (250–300 nm) are characteristic of π→π* electronic transitions and are commonly associated with flavonoids, phenolic acids, and other conjugated aromatic systems (Figure 1, highlighted in yellow) [21].

The peaks at 250 and 270 nm may indicate the presence of simple phenolic acids, such as gallic acid or caffeic acid derivatives. Similarly, previous studies on methanolic extracts of *Montanoa bipinnatifida* stems have reported absorption bands in the 240–280 nm range; these were attributed to C-glycosylated flavonoids, including luteolin and apigenin, consistent with the typical electronic transitions of these compounds [6].

Additionally, a low-intensity absorption band was observed between 390 and 410 nm (Figure 1, highlighted in aquamarine), within the transition region between ultraviolet and visible light. This band may be attributed to oxidized flavonoids, quinones, or partially degraded chlorophyll derivatives, all of which possess extended conjugated systems capable of absorbing in this spectral range [22,23,24]. Although specific absorption features in this region have not been reported for *M. bipinnatifida*, similar bands have been described in other species of the Asteraceae family and are commonly associated with oxidation products of flavonoids or stabilized plant pigments formed during extraction processes [25].

The presence of this band suggests the formation or preservation of complex chromophoric structures during maceration, which may be related to the biological activity of the extract. Comparable absorption features have been reported in *Spiraea japonica* (Rosaceae), with bands around 384–422 nm attributed to flavonoids with extended conjugation across the B and C rings [26].

Since the extract was obtained from leaves through ethanolic extraction, the signal observed at 657 nm, together with the absorption near 400 nm, can be primarily attributed to chlorophyll pigments and their derivatives [27]. This assignment aligns with the characteristic bands of tetrapyrrole compounds, particularly the Soret band in the 400–450 nm region and the Q band in the red region of the spectrum, around 650–670 nm. The most likely derivatives include chlorophyll *a*, chlorophyllide *a*, pheophytin *a*, and pheophorbide *a*, with a possible contribution from chlorophyll b derivatives, whose red absorption typically appears at slightly lower wavelengths (i.e., near 650–654 nm) [23,28]. The persistence of this band may also reflect the partial stability of these functional pigments following the maceration process.

Overall, the UV–Vis spectral profile of MB-LE indicates a chemically diverse composition, showing absorption features consistent with the conjugated systems and chromophoric groups commonly present in plant secondary metabolites. These characteristics may be related to the antioxidant potential of the extract; however, they should be regarded only as preliminary and non-specific indicators, as complex plant extracts such as MB-LE often exhibit overlapping signals from multiple compound classes. Consequently, the UV–Vis profile was employed as supplementary evidence to describe the general spectroscopic behavior of the extract, rather than as definitive proof of the presence of specific metabolite families.

### 3.3. FTIR Spectroscopic Analysis of MB-LE

**Figure 2:**
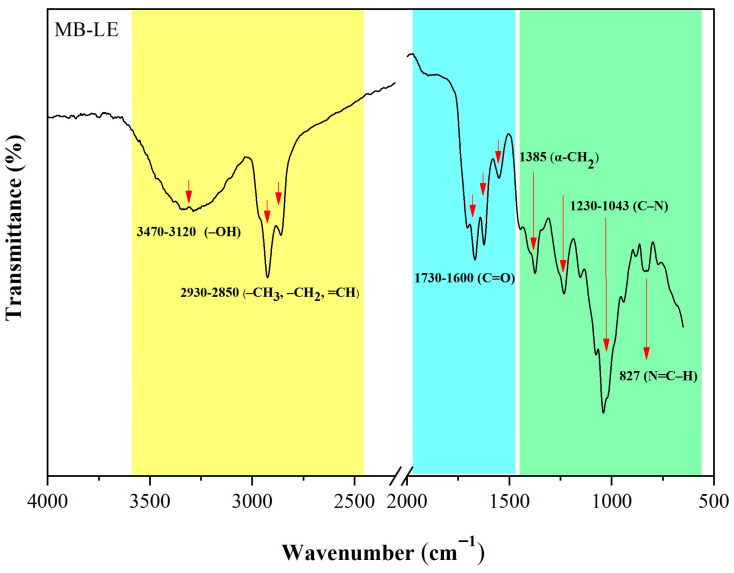
FTIR spectrum of MB-LE, showing absorption bands corresponding to O–H and C–H groups, characteristic of phenolics, lipids, and other secondary metabolites.

The FTIR spectrum of MB-LE exhibited characteristic absorption bands that enabled the identification of various functional groups (Figure 2).

The broad and intense band observed in the 3470–3120 cm^−1^ region is attributed to O–H stretching vibrations, typical of hydroxyl groups present in alcohols, phenols, and carboxylic acids. This feature is commonly associated with plant extracts that are rich in phenolic compounds and flavonoids [29].

The absorption bands detected in the 2930–2850 cm^−1^ region correspond to C–H stretching vibrations of methyl (–CH_3_), methylene (–CH_2_), and olefinic (=CH) groups. These signals are indicative of the presence of both saturated and unsaturated aliphatic chains, which are commonly found in lipids, terpenoids, and other secondary metabolites.

The absorption band at 1657 cm^−1^ is attributed to C=O stretching vibrations, which is characteristic of carbonyl groups presents in quinones–redox-active compounds that may contribute to the antioxidant properties of the extract. Additionally, bands observed within the 1600–1730 cm^−1^ region correspond to C=O stretching of various carbonyl-containing functional groups, including ketones, aldehydes, and carboxylic acids, confirming the presence of structurally diverse carbonyl compounds in MB-LE [30].

The band at 1385 cm^−1^ is assigned to C–H bending vibrations of methylene (–CH_2_) groups, indicative of the aliphatic hydrocarbon chains commonly found in lipids and related metabolites [31]. The absorption bands at 1230 and 1043 cm^−1^ are associated with C–N stretching vibrations, typical of amines and other nitrogen-containing compounds, suggesting the presence of amino acids, alkaloids, or nitrogenous glycosides within the extract [32].

Finally, the band observed at 827 cm^−1^ can be attributed to out-of-plane deformation vibrations of =C–H bonds. These are often associated with substituted aromatic or heterocyclic structures, which are common in nitrogen-containing secondary metabolites.

Due to the non-specific nature of this technique and the overlapping absorption bands typically observed in crude hydroalcoholic extracts such as MB-LE, these data were not employed to confirm the presence of metabolite classes. Instead, they are considered as complementary evidence of the extract’s overall chemical complexity, while more precise phytochemical assignments were derived from chromatographic analyses. In this context, the FTIR spectral profile of MB-LE revealed a chemically heterogeneous matrix, characterized by functional groups consistent with the plant secondary metabolites previously suggested by the chromatographic data, including phenolic compounds, glycosides, quinones, and nitrogen-containing metabolites. This compositional diversity underscores the potential of *M. bipinnatifida* as a reservoir of bioactive compounds with possible pharmacological relevance.

### 3.4. Phytochemical Profiling of the MB-LE via ESI-FT-ICR-MS

Untargeted metabolomic analysis of MB-LE enabled the comprehensive characterization of its phytochemical profile and the identification of major compound classes based on accurate mass measurements and relative signal intensities. This approach facilitated correlation between the bioactive properties traditionally attributed to this species in Mexican herbal medicine and the detected chemical constituents.

A total of 52 features in positive electrospray ionization mode (ESI+) and 289 features in negative mode (ESI−) were detected and subjected to molecular formula assignment based on high-resolution exact mass data. Filtering criteria included stringent mass accuracy thresholds and database matching. Table 1 and Table 2 summarize the most abundant compounds that met a mass error criterion of ≤5 ppm [33,34], while Supplementary Material S1 (Tables S1–S3) provides the complete set of molecular features within error tolerances of ≤5 ppm and ≤20 ppm.

It is important to note that the compound annotations reported herein are putative, as they are based on accurate mass measurements and database comparisons. Definitive structural confirmation would require complementary analyses, such as tandem mass spectrometry (MS/MS), isolation of individual compounds, and/or comparison with authentic reference standards.

The predominance of hypaphorine in ESI− (25.4%) represents the most pharmacologically relevant finding, consistent with preclinical evidence supporting its anti-inflammatory activity through modulation of the DUSP1/p38/JNK signaling pathway, as well as with omics-based studies associating this metabolite with anti-inflammatory profiles [42,57]. Its classification as an indole betaine is also consistent with its enhanced detectability under negative ionization conditions [58].

The co-detection of a steroidal saponin (Brodiosaponin A) suggests potential anti-inflammatory and antimicrobial properties, in agreement with the reported bioactivities for this class of compounds [59,60]. The presence of Brodiosaponin A should be interpreted with caution, as steroidal saponins are known to exert cytotoxic or hemolytic effects through interactions with cholesterol-rich membranes [61,62]. Nevertheless, its detection in the crude extract does not, by itself, demonstrate a toxicological risk, since such effects depend on multiple factors, including concentration, molecular structure, route of exposure, and compound bioavailability [63,64]. Given that the present analysis provides relative identification rather than absolute quantification, it cannot be concluded whether Brodiosaponin A would reach toxic concentrations in biological systems. Therefore, its presence is regarded as a precautionary signal that warrants further studies focused on quantification, cytotoxicity in non-tumor cell lines, and hemolytic activity.

The identification of K-Strophanthol-γ indicates the potential presence of a cardenolide capable of inhibiting Na^+^/K^+^-ATPase, a mechanism historically exploited in cardiovascular therapy. Nevertheless, its clinical applicability is currently limited due to its narrow therapeutic index and associated toxicity risks [65,66].

Similarly, the detection of a vincorine-type indole alkaloid is noteworthy given the diverse pharmacological activities reported for this family, including anticancer and neuroactive effects; however, any functional interpretation remains speculative in the absence of structural confirmation [67,68,69].

In contrast, signals corresponding to pseudoverdin and (+)-octopine may reflect metabolites of microbial origin, which introduces important considerations regarding extract composition, potential synergistic effects, and the reproducibility of the phytochemical profile [70,71,72]. These signals should be interpreted with caution as putative markers associated with microorganisms, and not as definitive evidence of endophytic colonization. Untargeted metabolomics data (ESI-FT-ICR-MS) alone do not allow us to distinguish whether these compounds originate from true endophytic microorganisms, epiphytic or environmental microorganisms present on the leaf surface, or incidental contamination during harvesting and processing [73]. Given that pseudoverdine/pyoverdine-type compounds are often associated with siderophore-producing bacteria, such as *Pseudomonas*, and octopine has been linked to plant–*Agrobacterium* interactions, their presence could indicate a microbial contribution to the chemical composition of the crude extract [74,75]. Future studies using surface-sterilized tissues, microbial isolation, 16S/ITS sequencing, and targeted confirmation via LC-MS/MS will be necessary to clarify the origin of such compounds and improve the reproducibility of the phytochemical profile [76].

Overall, the chemical profile of MB-LE established via FI-ESI-FT-ICR-MS reveals a structurally diverse metabolite composition with potential preclinical bioactivity. However, the putative nature of the annotations and the complexity of the extract highlight the need for targeted isolation, structural validation, and bioactivity-guided assays to establish clear relationships between specific metabolites and their biological effects.

### 3.5. Gas Chromatography–Mass Spectrometry Analysis of MB-LE

**Figure 3:**
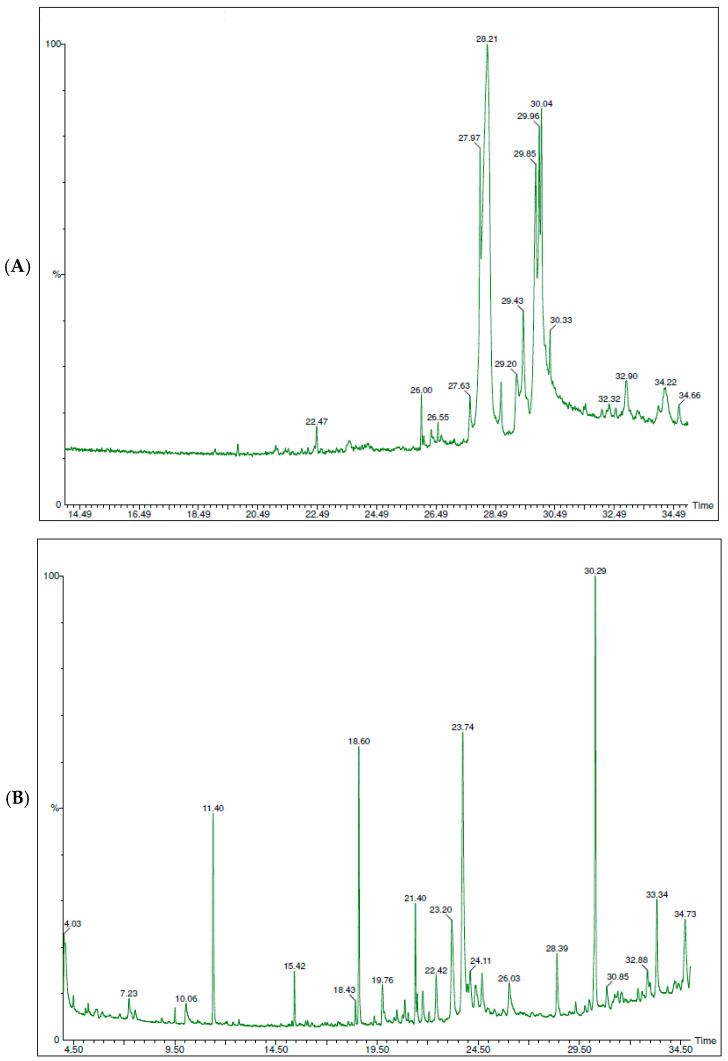
GC–MS chromatograms of MB-LE before and after derivatization. (**A**) Non-derivatized sample, showing predominance of late-eluting, less volatile compounds. (**B**) Derivatized sample, with increased peak numbers, especially at lower retention times, due to enhanced volatility.

The chemical composition of MB-LE was further characterized using gas chromatography–mass spectrometry (GC–MS). The resulting chromatographic profiles are presented in Figure 3A,B, while a comprehensive list of the identified compounds is provided in Supplementary Material S2 (Tables S4 and S5).

**Figure 4:**
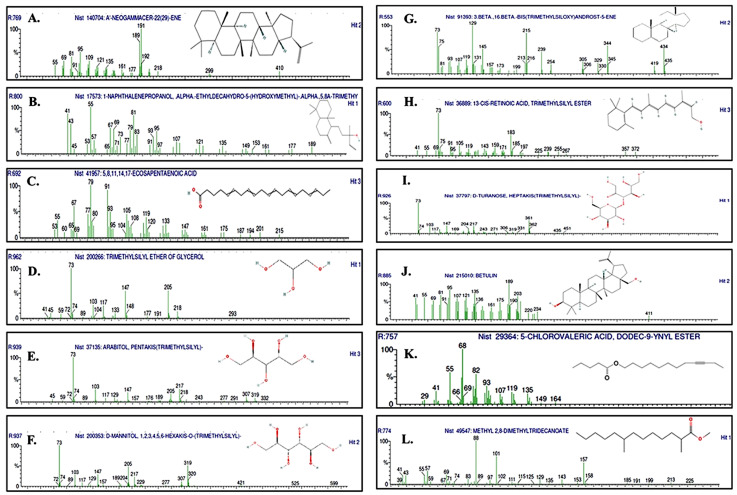
Mass spectra of selected compounds identified in the MB-LE extract: (**A**) A′-Neogammacer-22(29)-ene; (**B**) 1-naphthalenepropanol, α-ethyldecahydro-5-(hydroxymethyl)-α,5,8a-trimethyl; (**C**) 5,8,11,14,17-eicosapentaenoic acid; (**D**) trimethylsilyl ether of glycerol; (**E**) arabitol, pentakis(trimethylsilyl); (**F**) D-mannitol, 1,2,3,4,5,6-hexakis-O-(trimethylsilyl); (**G**) 3β,16β-bis(trimethylsiloxy)androst-5-ene; (**H**) 13-cis-retinoic acid, trimethylsilyl ester; (**I**) D-turanose, heptakis(trimethylsilyl); (**J**) betulin; (**K**) 5-chlorovaleric acid, dodec-9-ynyl ester; and (**L**) methyl 2,8-dimethyltridecanoate. Identifications were based on comparison with the NIST library.

For clarity and interpretability, the following discussion focuses on the most abundant constituents selected based on their relative peak area percentages, as summarized in Table 3 and Figure 4.

The major constituents identified encompass a diverse range of chemical classes, including terpenes, polyols, retinoids, fatty acids, steroids, and sugar derivatives. Among the most prominent terpenoid compounds were A′-neogammacer-22(29)-ene (also reported as manool-type diterpenoid derivatives), 1-naphthalenepropanol, α-ethyl-decahydro-5-(hydroxymethyl)-α,5,8a-trimethyl derivatives, and betulin.

Manool—a labdane-type diterpenoid—has been widely reported to exhibit a broad spectrum of bioactivities, including antineoplastic, anti-inflammatory, antibacterial, antiviral, and antimycobacterial effects [77,78,79]. Similarly—betulin a lupane-type triterpene—has demonstrated multiple pharmacological properties, such as selective anticancer, anti-inflammatory, antioxidant, neuroprotective, and antiviral activities [80,81,82].

Several sugar alcohols were identified, including glycerol, arabitol, and D-mannitol. These polyols are widely used in food and pharmaceutical formulations, and are known to exhibit osmotic, anticariogenic, antibacterial, and glucose-regulating properties [83,84].

Additionally, 5, 8, 11, 14, 17-eicosapentaenoic acid (EPA)—an omega-3 polyunsaturated fatty acid—was detected. EPA has been extensively studied for its anti-inflammatory, neuroprotective, antidepressant, and cardioprotective effects, and it serves as a precursor to antithrombotic prostanoids [85,86].

Among the steroidal constituents, androst-5-ene, a compound recognized as a testosterone prohormone with reported anti-inflammatory, antiproliferative, and metabolic-modulating activities, was identified [87,88,89]. Retinoids were represented by 13-cis-retinoic acid, a bioactive molecule with established clinical applications in the treatment of acne, psoriasis, and certain cancers, primarily due to its ability to regulate gene expression and modulate inflammatory responses [90,91].

Other relevant constituents include D-turanose—a sucrose isomer with reported cryoprotective, anticariogenic, and stabilizing properties—as well as two compounds with limited available experimental evidence: 5-chlorovaleric acid, dodec-9-ynyl ester; and methyl 2,8-dimethyltridecanoate [92].

Overall, the GC–MS analysis demonstrates that MB-LE contains a structurally diverse array of bioactive metabolites, many of which are consistent with the antioxidant and anti-inflammatory activities previously reported for species of the *Montanoa* genus. These findings provide additional chemical support for the observed biofunctional properties of the extract and highlight promising candidates for further pharmacological investigation.

### 3.6. Antioxidant Activity

Antioxidant compounds can act through multiple chemical mechanisms, including hydrogen atom transfer (HAT), single-electron transfer (SET), and transition metal chelation. Due to this mechanistic diversity, the use of multiple complementary assays is necessary to carry out a comprehensive evaluation of the antioxidant potential of complex plant extracts. Moreover, the activity of phytochemicals depends not only on their chemical structure but also on the assay conditions, solvent system, and nature of the radical or oxidant species employed [93].

In this study, the antioxidant capacity of MB-LE was assessed using three widely accepted and complementary methods: DPPH, ABTS, and FRAP.

#### 3.6.1. DPPH Radical Scavenging Activity

**Figure 5:**
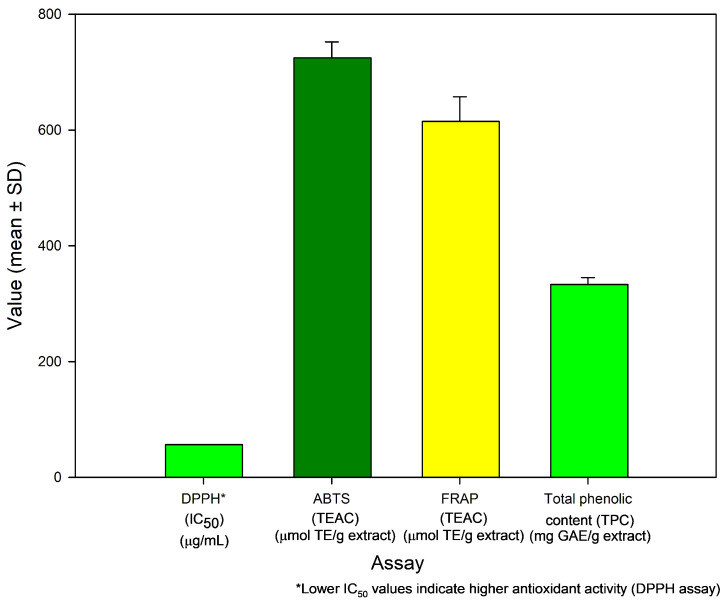
Antioxidant capacity and total phenolic content of the MB-LE extract. Values are expressed as mean ± SD (*n* = 3). Lower IC_50_ values indicate higher antioxidant activity in the DPPH assay. Values are expressed in different units and are not directly comparable. The different assays reflect distinct antioxidant mechanisms, including hydrogen atom transfer (HAT) and single-electron transfer (SET).

The DPPH assay revealed that MB-LE exhibits moderate radical scavenging activity, with an IC_50_ value of 56.21 ± 0.67 µg/mL (Figure 5). This parameter reflects the concentration required to reduce 50% of the DPPH radical signal and is commonly used as an indicator of the hydrogen- or electron-donating capacity of antioxidant compounds. The DPPH assay is particularly sensitive to lipophilic antioxidants due to the solubility of the radical in organic media [94].

The antioxidant activity detected via DPPH assay is primarily associated with compounds capable of donating hydrogen atoms or electrons, such as phenolic acids, flavonoids, and other polyphenols. The presence of hydroxyl groups in these molecules enhances their ability to stabilize free radicals through resonance, thereby interrupting oxidative chain reactions.

The IC_50_ value obtained indicates moderate antioxidant activity when compared to pure reference compounds such as Trolox, gallic acid, or quercetin, which typically exhibit IC_50_ values in the range of 5–20 µM under similar experimental conditions [95]. The comparatively lower activity of MB-LE can be attributed to its nature as a crude extract, comprising a complex mixture of metabolites with varying and potentially synergistic or antagonistic redox properties.

Consistent with these findings, moderate antioxidant activity has been reported in other species of the *Montanoa* genus, including *M. tomentosa* and *M. frutescens*, in which flavonoids, phenolic acids, and terpenoids have been identified as the main contributors to bioactivity [6,96]. This supports the hypothesis that similar classes of compounds are responsible for the observed activity of MB-LE.

Comparatively, Vásquez-Cardeño et al. (2007) [97] reported lower antioxidant activity in *Montanoa ovalifolia* leaf extracts relative to other medicinal species. In this context, the IC_50_ obtained for *M. bipinnatifida* suggests a relatively higher radical scavenging capacity within the genus.

From a broader phytochemical perspective, species within the Asteraceae family such as *Arnica montana* and *Artemisia absinthium* have demonstrated comparable DPPH scavenging activities. These species are known to contain phenolic acids (e.g., gallic, chlorogenic, caffeic, and ferulic acids) and flavonoids such as quercetin, rutin, and luteolin, which are well-established antioxidants [98,99,100]. The similarity in antioxidant performance suggests that MB-LE may share analogous bioactive constituents.

Likewise, *Taraxacum officinale* (dandelion)—another Asteraceae species rich in polyphenols and flavonoids—has shown IC_50_ values in a similar range when evaluated under comparable conditions [101]. This further supports the relevance of *M. bipinnatifida* as a potential source of natural antioxidants.

#### 3.6.2. ABTS Radical Cation Scavenging Activity

The antioxidant capacity of MB-LE evaluated via the ABTS assay showed a value of 724.82 ± 27.49 µmol Trolox equivalents per g of extract (Figure 5). This assay is based on the reduction in the pre-formed ABTS^•+^ radical cation, monitored as a decrease in absorbance at 734 nm, and is particularly advantageous due to its applicability in both aqueous and organic systems, allowing for the assessment of both hydrophilic and lipophilic antioxidants [102].

In contrast to DPPH, which is more sensitive to lipophilic compounds, the ABTS assay provides a broader estimation of antioxidant capacity by capturing contributions from a wider range of molecular species. The response observed for MB-LE therefore suggests that both polar and moderately non-polar constituents participate in its overall redox activity.

The magnitude of the response is consistent with a moderate antioxidant capacity, in agreement with the DPPH results. However, it is important to note that direct comparisons with IC_50_ values of pure standards such as Trolox are not strictly appropriate, as the ABTS result is expressed in Trolox equivalents (TEAC) rather than inhibitory concentration. Instead, TEAC values should be interpreted comparatively across extracts analyzed under similar conditions.

When expressed on a per gram basis, the antioxidant capacity of MB-LE appears to be higher than that for ethanolic extracts of *Arnica montana* and *Artemisia absinthium*, for which values of 486.06 and 690.62 µmol Trolox/g extract were reported, respectively [100]. This highlights the importance of unit consistency and normalization when comparing antioxidant data across studies.

Similarly, *Centaurea albonitens* has been reported to exhibit lower ABTS activity (~95.99 µmol Trolox/g extract) [103], reinforcing that the antioxidant capacity of MB-LE falls within the mid-to-high range for Asteraceae species.

The differences observed between ABTS and DPPH responses can be attributed to the chemical heterogeneity of the extract. In complex matrices such as MB-LE, antioxidant performance is governed not only by total phenolic content but also by structural features (e.g., hydroxylation patterns, conjugation), solubility, and steric accessibility to the radical species. Additionally, the ABTS assay tends to overestimate the contribution of highly reactive hydrophilic antioxidants, which may explain the relatively higher values obtained compared to DPPH [104].

From a phytochemical standpoint, previous reports on *Montanoa bipinnatifida* have identified C-glycosylated flavonoids such as luteolin and apigenin derivatives, which are known to exhibit strong antioxidant activity via both electron transfer and hydrogen atom donation mechanisms [6]. The presence of these compounds, together with other phenolics and terpenoids described in the genus, provides a plausible chemical basis for the activity observed in MB-LE.

Overall, the ABTS results reinforce the notion that MB-LE is characterized by a chemically diverse antioxidant system, with contributions from multiple classes of metabolites acting through complementary redox mechanisms.

#### 3.6.3. Ferric Reducing Antioxidant Power (FRAP)

The antioxidant capacity of MB-LE was further evaluated using the FRAP assay, yielding a value of 614.90 ± 42.63 µmol Trolox equivalents per g of extract (Figure 5). This method is based on the reduction in ferric ions (Fe^3+^) to ferrous ions (Fe^2+^) under acidic conditions, leading to the formation of a Fe^2+^–TPTZ complex with characteristic absorbance at 593 nm [105].

The FRAP response reflects the electron-donating capacity of the sample, and is therefore particularly sensitive to reductive antioxidants such as phenolic acids and flavonoids. The presence of these compounds in MB-LE, as suggested by the spectroscopic and metabolomic analyses, likely contributes significantly to its observed reducing power. Structural features such as hydroxylation patterns and conjugated systems facilitate electron transfer processes, thus stabilizing oxidized intermediates.

The magnitude of the FRAP value indicates a moderate-to-high reducing capacity, in agreement with the trends noted for the DPPH and ABTS assays. When compared with other Asteraceae species, MB-LE falls within the reported range; for example, previous studies have reported values for *Parastrephia lucida* and *Leonurus javanicus* of 255.50 and 799.01 µmol Trolox/g dry weight, respectively [106,107], situating MB-LE in an intermediate position.

It is important to emphasize that the FRAP assay is selective for single-electron transfer (SET) mechanisms and does not adequately capture antioxidant activity mediated by hydrogen atom transfer (HAT) or radical quenching via steric or kinetic effects. Moreover, although some compounds capable of metal chelation may contribute indirectly, FRAP does not directly measure chelating activity. Therefore, the results should be interpreted as part of a complementary analytical framework rather than as a standalone indicator of total antioxidant capacity [108].

In contrast with DPPH and ABTS, reports on FRAP activity within the *Montanoa* genus remain scarce, limiting direct comparisons. Furthermore, when contrasting MB-LE with other plant systems, caution is required, for example, Nowak et al. (2019) reported FRAP values for *Taraxacum officinale* extracts expressed as mg Trolox/g raw material [109], whereas the present study expresses results in µmol Trolox/g extract. These differences in units, extraction yields, and sample normalization preclude direct quantitative comparison, and may lead to overestimation of relative antioxidant strength if not properly standardized.

Overall, the FRAP results support the presence of a chemically diverse set of reductive antioxidants in MB-LE, likely dominated by phenolic constituents such as flavonoids (e.g., luteolin and apigenin derivatives) and phenolic acids. These compounds, which have previously been reported in *Montanoa* species, provide a plausible mechanistic basis for the observed electron transfer capacity.

### 3.7. Total Phenolic Content (TPC)

The mean total phenolic content (TPC) of MB-LE was 333.31 ± 11.93 mg gallic acid equivalents (GAE)/g of dry extract (Figure 5), indicating a high abundance of reducing compounds within the extract. This result is consistent with the antioxidant capacity observed across the DPPH, ABTS, and FRAP assays, supporting the contribution of phenolic constituents to the overall redox activity.

When compared with other members of the Asteraceae family, MB-LE exhibits a markedly higher TPC, for instance, methanolic leaf extracts of *Petasites hybridus* have been reported to contain between 4.43 and 19.92 mg GAE/g dry weight [110], placing MB-LE among the phenolic-rich plant extracts.

Phenolic compounds are well known for their ability to donate electrons or hydrogen atoms and to stabilize radical species through resonance delocalization. Therefore, their high abundance in MB-LE likely plays a central role in the antioxidant responses observed. However, it is important to interpret TPC values with caution, as the Folin–Ciocalteu assay is not entirely specific to phenolics and can also respond to other reducing agents, including ascorbic acid, certain sugars, and nitrogen-containing compounds [111]. Consequently, TPC should be considered an estimate of total reducing capacity rather than an absolute quantification of phenolic compounds.

## 4. Discussion

### 4.1. Traditional Uses, Proposed Mechanisms of Action, and Potential Applications of MB-LE

The phytochemical profile of MB-LE, characterized by the presence of flavonoids, phenolic acids, terpenes, fatty acids, steroids, retinoids, and polyols, provides a plausible biochemical basis for its traditional use in Mexican medicine, particularly in wound healing, analgesia, and anti-inflammatory applications.

From a mechanistic perspective, the antioxidant components of MB-LE may contribute to the modulation of oxidative stress by scavenging reactive oxygen species (ROS) generated during tissue injury, which can help limit oxidative damage to biomolecules such as lipids, proteins, and DNA [112,113]. Additionally, certain phytochemicals may indirectly support endogenous antioxidant defenses (e.g., SOD, CAT, GPx) through redox-sensitive signaling pathways, although this effect should be interpreted cautiously in the absence of direct enzymatic assays [114].

The anti-inflammatory potential of the extract may be associated with the modulation of key mediators such as TNF-α, IL-1β, and other cytokines, facilitating the transition from the inflammatory to the proliferative phase of wound healing [115,116]; however, these mechanisms remain hypothetical in the context of the present study and should be validated experimentally.

Specific metabolites identified in MB-LE provide additional insight into its potential bioactivity. For example, eicosapentaenoic acid (EPA) has been associated with anti-inflammatory and cytoprotective effects, partly through the modulation of lipid mediators and mitochondrial function [117,118,119]. Retinoids such as 13-cis-retinoic acid are known regulators of cell differentiation, immune responses, and tissue remodeling, although their biological effects are highly dose-dependent and context-specific [120,121,122].

Betulin—one of the most pharmacologically relevant triterpenes identified—has been widely reported to modulate inflammatory signaling pathways such as NF-κB and MAPK, while also activating cytoprotective responses mediated by Nrf2. These effects are associated with reduced expression of pro-inflammatory mediators and enhanced antioxidant defense [123,124,125,126]. Additionally, betulin derivatives have demonstrated pro-regenerative effects in skin models, including stimulation of keratinocyte proliferation and collagen synthesis [127,128,129,130]. Notably, a betulin-based oleogel (Episalvan^®^) has been clinically approved for wound healing applications, supporting the translational relevance of this compound [131].

From an application standpoint, the chemical composition of MB-LE suggests its potential for utilization across multiple sectors. In the food industry, the extract could serve as a natural antioxidant to delay lipid oxidation and improve the stability of fat-containing systems. Its incorporation into edible coatings or active packaging materials may also contribute to shelf-life extension of perishable products [132,133,134,135].

In pharmaceutical and dermatological contexts, MB-LE or its enriched fractions could be explored as components of topical formulations (e.g., gels, creams, ointments) aimed at wound care or inflammatory skin conditions. However, it is essential to emphasize that the presence of bioactive compounds does not guarantee efficacy or safety in clinical settings, and further studies—including toxicity, bioavailability, and dose—response evaluations—are required.

Finally, although compounds such as retinoids and steroid derivatives suggest possible dermatological applications (e.g., acne treatment), such extrapolations should be approached with caution due to the complexity of extract matrices and the potential for adverse effects associated with these molecules at pharmacological doses.

### 4.2. Total Phenolic Content

The extract showed a high total phenolic content (333.31 ± 11.93 mg GAE/g), supporting the contribution of phenolic compounds to its antioxidant profile. However, it should be noted that the Folin–Ciocalteu assay is not entirely specific and may also detect other reducing substances. Therefore, the values obtained should be interpreted as an estimate of total reducing capacity rather than the absolute phenolic concentration.

### 4.3. Biological Relevance and Potential Applications

The phytochemical composition and antioxidant profile of MB-LE are consistent with its traditional use in wound-related conditions. The presence of compounds with reported anti-inflammatory and redox-modulating properties suggests a plausible mechanistic basis. Nevertheless, these interpretations remain hypothesis-driven, as the present study did not include direct biological validation.

From an applied perspective, the extract may represent a promising source of natural antioxidants for food, cosmetic, or pharmaceutical formulations; however, further studies addressing bioavailability, toxicity, and efficacy concerns are required before practical applications can be considered.

## 5. Conclusions

A hydroethanolic extract (70% ethanol) of *Montanoa bipinnatifida* leaves (MB-LE) was successfully obtained via maceration, efficiently recovering polar and semipolar metabolites. Spectroscopic analyses (UV–Vis and FTIR) confirmed the presence of phenolic compounds, flavonoids, quinones, and nitrogen-containing functional groups, reflecting the chemical complexity of the extract.

High-resolution metabolomic profiling (ESI-FT-ICR-MS), complemented by GC–MS analysis, revealed a diverse array of secondary metabolites, including alkaloids, saponins, terpenoids, and fatty acids. While these identifications remain putative, the detection of metabolites such as hypaphorine, betulin, and eicosapentaenoic acid suggests potential contributions to antioxidant and anti-inflammatory processes, consistent with previous reports for other *Montanoa* species.

Functionally, MB-LE exhibited moderate antioxidant activity in the DPPH, ABTS, and FRAP assays, accompanied by a high total phenolic content (TPC). These results highlight the central role of phenolic compounds in redox activity and suggest that multiple constituents within the crude extract present additive or synergistic effects.

Overall, this study broadens the phytochemical understanding of *M. bipinnatifida* leaves and provides a preliminary chemical and functional characterization of MB-LE. Nonetheless, the proposed biological relevance of the detected metabolites should be regarded as hypothesis-generating, since several assignments and potential activities were based on putative annotations and literature-derived evidence. Future investigations should prioritize structural confirmation and targeted quantification of key metabolites, alongside the evaluation of biological activity in relevant in vitro and in vivo models, as well as safety, bioavailability, and reproducibility assessments. Such efforts will be essential to validate the contribution of individual metabolites and determine the potential of MB-LE as a source of bioactive compounds for nutraceutical, pharmaceutical, or related applications.

## Figures and Tables

**Table 1:** Abundant phytochemicals identified in MB-LE via FI-ESI-FT-ICR-MS in negative ionization mode (ESI−).

*m*/*z*	Metabolite Annotation *	Formula	Peak Abundance ^a^	Relative Abundance ^a^	Class	Reported Biological Activities	Ref.
245.12276	(+)-Octopine	C_9_H_18_N_4_O_4_	2.52 × 10^8^	6.37	Opine	Antitumor, antioxidant, hypocholesterolemic	[35,36]
246.12804	12-Cytisineacetamide	C_13_H_17_N_3_O_2_	5.84 × 10^7^	1.47	Quinolizidine alkaloid	Neuroprotective, anti-inflammatory, antimicrobial	[37,38,39,40,41]
245.12049	DL-Hypaphorine	C_14_H_18_N_2_O_2_	1.01 × 10^9^	25.40	Betaine-type indole alkaloid	Anti-inflammatory, antihyperglycemic, antiviral	[42,43,44,45,46,47]
247.13075	Diptocarpamine	C_11_H_24_N_2_O_2_S	9.28 × 10^7^	2.34	Indole alkaloid	Cytotoxic, antimicrobial	[48]
245.12403	Ligudentatin A	C_15_H_18_O_3_	2.12 × 10^8^	5.36	Quinolizidine alkaloid	Anti-inflammatory, respiratory effects	[49]
245.10702	Prolyl-Methionine	C_10_H_18_N_2_O_3_S	1.78 × 10^8^	4.50	Oligopeptide	Antioxidant, antihypertensive, anti-inflammatory	[50,51,52]

* Metabolite annotations are putative and based on accurate mass and database matching. Structural confirmation requires MS/MS fragmentation and/or authentic standards. ^a^ Peak abundance and relative abundance correspond to detected ion intensity in ESI− mode.

**Table 2:** Abundant phytochemicals identified in MB-LE via FI-ESI-FT-ICR-MS in positive ionization mode (ESI+).

*m*/*z*	Metabolite Annotation *	Formula	Peak Abundance *	Relative Abundance ^a^	Class	Reported Biological Activities	Ref.
805.4186	Vincorine-type bisindole alkaloid	C_47_H_56_N_4_O_8_	3.25 × 10^6^	3.20	Monoterpenoid indole alkaloid	Antitumor, antiproliferative	[53]
919.53652	Brodiosaponin A	C_44_H_70_O_20_	3.89 × 10^6^	3.83	Triterpene Saponin	Anti-inflammatory, antimicrobial	[54]
875.51112	K-Strophanthol-γ	C_42_H_66_O_19_	7.19 × 10^6^	7.08	Cardiotonic glycoside	Na^+^/K^+^-ATPase inhibitor, antiviral	[55]
222.02099	Pseudoverdin	C_10_H_7_NO_5_	5.01 × 10^6^	4.93	Coumarin derivative	Antibacterial, antitumor	[56]

* Metabolite annotations are putative and based on accurate mass and database matching. Structural confirmation requires MS/MS fragmentation and/or authentic standards. ^a^ Peak abundance and relative abundance correspond to detected ion intensity in ESI+ mode.

**Table 3:** Representative mass spectra of the major compounds * identified in the MB-LE via GC–MS analysis.

Volatile Compounds	Non-Volatile Compounds ^α^
A′-Neogammacer-22(29)-ene (manool)	Glycerol (Trimethylsilyl ether)
5,8,11,14,17-Ecosapentaenoic acid (EPA)	Arabitol (pentakis-TMS)
1-Naphthalenepropanol derivate	D-mannitol (hexakis-TMS)
5-Chlorovaleric acid derivate	Androst-5-ene derivate
Methyl 2,8-dimethyltridecanoate	13-cis-Retinoic acid (TMS ester)
-	D-Turanose (heptakis-TMS)
-	Betulin

* Compounds were tentatively identified through comparison of mass spectra with spectral libraries. ^α^ TMS: trimethylsilyl derivatives obtained after derivatization.

## Data Availability

The original contributions presented in the study are included in the article. Further inquiries can be directed to the corresponding author.

## Supplementary Materials

The following supporting information can be downloaded at https://www.mdpi.com/article/10.3390/antiox15050598/s1: Supplementary Material (S1) Tables S1–S3. FI-ESI FTICR-MS analysis; Supplementary Material (S2) Tables S4 and S5. GC-MS Analysis.

## Abbreviations

The following abbreviations are used in this manuscript:

MB-LE	*Montanoa bipinnatifida* C. Koch leaf extract
FTIR	Fourier Transform Infrared spectroscopy
ESI-FT-ICR-MS	Fourier transform ion cyclotron resonance coupled with electrospray ionization
GC-MS	Gas Chromatography coupled with Mass Spectrometry
UV–Vis	Ultraviolet-visible spectroscopy
TNF-α	Tumor Necrosis Factor α
IL-6	Interleukin-6
EPA	Eicosapentaenoic Acid
IL-1β	Interleukin-1 beta
NF-κB	Nuclear Factor kappa-light-chain-enhancer of activated B cells
MAPK	Mitogen-Activated Protein Kinase
DPPH	2,2-diphenyl-1-picrylhydrazyl
Na_2_CO_3_	Sodium carbonate
ATR	Attenuated Total Reflectance
MeOH	Methanol
ABTS^•+^	2,2′-azino-bis (3-ethylbenzothiazoline-6-sulfonic acid)
TAC	Total antioxidant content
Na_2_S_2_O_8_	Sodium persulfate
FRAP	Ferric ion Reducing Antioxidant Power
Fe^3+^-TPTZ	Ferric ions (Fe^3+^) with the ligand 2,4,6-tri(2-pyridyl)-s-triazine
Fe^2+^-TPTZ	Ferrous ions (Fe^2+^) with the ligand 2,4,6-tri(2-pyridyl)-s-triazine
EAG	Gallic Acid Equivalent
H_2_O	Water
PVDF	Polyvinylidene fluoride
IC_50_	Half-maximal Inhibitory Concentration
TPC	Total Phenolic Content
NIST	National Institute of Standards and Technology
