# Supplementary material for: Phytochemical Profile and Antioxidant Potential of Montanoa bipinnatifida C. Koch Leaf Extract: Promising Bioactives for Pharmaceutical Applications
Source: Antioxidants (Basel). 2026 May 8;15(5):598. doi: 10.3390/antiox15050598 (PMC13203521; doi:10.3390/antiox15050598)
Supplement: Supplementary file 1 [file antioxidants-15-00598-s001.zip › Suplemmentary material (S1) FI-ESI FTICR-MS.pdf]

SUPLEMENTARY MATERIAL (S1)

Table S1. Phytochemicals peaks identified in MB-LE via FI-ESI-FT-ICR MS analysis in positive electrospray ionization mode (ESI+, ≤5 ppm).

m/z	Metabolite annotation*	Molecular Formula	Peak Abundance$^{\alpha}$	% Relative Abundance$^{\alpha}$
795.48672	(3beta,14beta)-3,14-dihydroxy-21-methoxypregn-5-en-20-one-3-O-beta-oleandropyranosyl-(1->4)-O-beta-cymaropyranosyl-(1->4)-O-beta-cymaropyranoside∣perisepiumoside E	$C_{43}H_{70}O_{13}$	1.17E+06	1.15
353.26747	(E)-7-hexadecenylitaconic acid∣ceriporic acid D	$C_{21}H_{36}O_4$	1.10E+06	1.08
409.1854	1-[3-(2-dimethylaminoethyl)-5-hydroxy-1h-4-indolyl]-3-hydroxy-3-(2-methylaminoethyl)-2-indolinone	$C_{23}H_{28}N_4O_3$	1.59E+06	1.57
637.39366	12beta-Hydroxycimigenol-3-O-alpha-L-arabinopyranoside	$C_{35}H_{56}O_{10}$	2.63E+06	2.59
773.49226	19'-Hexanoyl-19'-Hydroxyfucoxanthin	$C_{48}H_{68}O_8$	1.60E+06	1.58
257.24888	2,4b-Dimethyl-8-methylene-2-vinyl-1,2,3,4,4a,4b,5,6,7,8,8a,9-dodecahydrophenanthrene #	$C_{19}H_{28}$	2.04E+06	2.01
533.23954	2,7-Dihydroxy-2,8-dimethyl-4-vinyl-11-(2,8-dimethyl-7-hydroxy-9,10-dihydrophenanthrene-3-yl)-1,4-ethano-1,2,3,4,9,10-hexahydrophenanthrene-3-one	$C_{36}H_{36}O_4$	1.45E+06	1.43
889.04324	3-Methylglutaconyl-Coa	$C_{27}H_{37}N_7O_{19}P_3S$	2.11E+06	2.08
737.44527	3-O-beta-D-xylopyranosyl(1->3)-alpha-L-arabinopyranosylhederagenin∣3-O-[beta-D-xylopyranosyl-(1->3)-alpha-L-arabinopyranosyl]-hederagenin∣hederagenin 3-O-beta-D-xylopyranosyl-(1->3)-alpha-L-arabinopyranoside∣Hederagenin-3-O-??-D-xyl(1-3)-L-arabinopyranoside	$C_{40}H_{64}O_{12}$	1.30E+06	1.28
888.15895	3-hydroxybenzoyl-CoA	$C_{28}H_{40}N_7O_{18}P_3S$	1.11E+06	1.10
673.341	3beta,14beta,15beta-trihydroxypregn-5-en-20-one 3-O-beta-D-glucopyranosyl-(1->2)-beta-D-glucopyranoside∣cynaforroside J	$C_{33}H_{52}O_{14}$	2.06E+06	2.03
715.44797	4'-deoleandrosyl-6,8a-seco-6,8a-deoxyavermectin B1a	$C_{41}H_{62}O_{10}$	1.03E+06	1.01
381.08114	5,8-dihydroxy-5-O-beta-D-glucosyllpsoralen	$C_{17}H_{16}O_{10}$	2.46E+06	2.42
331.13277	6-O-Nicotinoylstrychnovoline	$C_{17}H_{18}N_2O_5$	1.13E+06	1.11

805.4186	Ac-11-[10-(11-Methoxyvincamajinyl)]vincorine	$C_{47}H_{56}N_4O_8$	3.25E+06	3.20
717.36815	Aldgamycin C	$C_{35}H_{56}O_{15}$	2.71E+06	2.67
977.64506	Apramide A	$C_{52}H_{80}N_8O_8S$	1.14E+06	1.12
963.56597	Apramide B	$C_{51}H_{78}N_8O_8S$	2.21E+06	2.17
577.25661	Blestritin A	$C_{37}H_{36}O_6$	1.09E+06	1.07
919.53652	Brodiosaponin A	$C_{44}H_{70}O_{20}$	3.89E+06	3.83
871.71385	Halicylindroside A3	$C_{50}H_{98}N_2O_9$	1.25E+06	1.23
977.13053	Hydroxyfucodiphlorethol B undecaacetate	$C_{46}H_{40}O_{24}$	1.33E+06	1.31
875.51112	K-Strophanthol-gamma	$C_{42}H_{66}O_{19}$	7.19E+06	7.08
919.60049	Megalomicin B; 4'-Acetylmegalomycin A	$C_{46}H_{82}N_2O_{16}$	1.26E+06	1.24
832.03464	Methylacrylyl-Coa	$C_{25}H_{36}N_7O_{17}P_3S$	1.62E+06	1.59
787.46054	Myrmekioside B	$C_{37}H_{70}O_{17}$	8.87E+06	8.73
802.51409	Phosphatidylethanolamine alkenyl 20:1-22:6	$C_{47}H_{80}NO_7P$	1.63E+06	1.60
883.53495	Phosphatidylinositol 16:0-22:6	$C_{47}H_{79}O_{13}P$	1.65E+06	1.62
867.54168	Phosphatidylinositol alkyl 16:1-22:6	$C_{47}H_{79}O_{12}P$	1.49E+06	1.47
832.5402	Phosphatidylserine 18:1-22:6	$C_{47}H_{78}NO_9P$	1.07E+06	1.05
222.02099	Pseudoverdin	$C_{10}H_7NO_5$	5.01E+06	4.93
925.52086	Rotundioside O	$C_{48}H_{76}O_{17}$	1.90E+06	1.87
969.6661	Salinixanthin	$C_{61}H_{92}O_9$	1.20E+06	1.18
890.07347	Sulfoacetyl-CoA	$C_{23}H_{38}N_7O_{20}P_3S_2$	1.13E+06	1.11
331.26217	Undecenyl phenanthrene	$C_{25}H_{30}$	1.40E+06	1.38
365.15876	Urea, tetraphenyl-	$C_{25}H_{20}N_2O$	1.75E+06	1.73
541.2631	Valtrate hydrine B3	$C_{27}H_{40}O_{11}$	1.17E+06	1.15
803.01422	Vitilagin	$C_{34}H_{26}O_{23}$	1.36E+06	1.34
861.05476	[(2S,3R,4S,5R,6R)-5-hydroxy-2,4-bis[(3,4,5-trihydroxybenzoyl)oxy]-6-[(3,4,5-trihydroxybenzoyl)oxymethyl]oxan-3-yl] 3,4,5-trihydroxy-2-(2-methoxy-2-oxoethyl)benzoate	$C_{37}H_{32}O_{24}$	1.26E+06	1.24
523.3042	Euphoractin L	$C_{32}H_{42}O_6$	1.53E+06	1.51
277.17859	Expansiol	$C_{17}H_{24}O_3$	1.30E+06	1.28

889.50311	Inundoside-D tetraacetate∣inundoside-D1 tetraacetate	$C_{52}H_{72}O_{12}$	1.86E+06	1.83
726.48032	Kailuin B	$C_{37}H_{67}N_5O_9$	1.13E+06	1.11
321.13281	Liguhodgcins A	$C_{14}H_{21}ClO_6$	1.06E+06	1.05
795.54588	Lucidanin	$C_{48}H_{74}O_9$	1.43E+06	1.41
985.64281	Majusculamide C	$C_{50}H_{80}N_8O_{12}$	5.35E+06	5.26
983.62294	Mycapolyol C	$C_{51}H_{86}N_2O_{16}$	1.03E+06	1.02
650.31897	Nigellamine A1	$C_{40}H_{43}NO_7$	2.24E+06	2.20
884.54219	Rubellidin 4.3	$C_{41}H_{73}N_9O_{12}$	1.01E+06	0.99
911.63066	trans,octacis-Decaprenylphospho-beta-D-ribofuranose	$C_{55}H_{91}O_8P$	1.40E+06	1.37
919.09462	Vescalagin	$C_{41}H_{26}O_{25}$	1.62E+06	1.59

* All metabolite annotations are putative and were assigned based on accurate mass and database matching. Structural confirmation requires MS/MS fragmentation and/or authentic standards.

[α]Total sum of peak abundance and % relative abundace of these compounds in the positive ESI mode.

Table S2. Phytochemicals peaks identified in MB-LE via FI-ESI-FT-ICR MS analysis in negative electrospray ionization mode (ESI-, ≤5 ppm).

m/z	Metabolite annotation*	Molecular Formula	Peak Abundance$^{\alpha}$	% Relative Abundance$^{\alpha}$
245.12276	(+)-Octopine	$C_9H_{18}N_4O_4$	2.52E+08	6.37
235.13803	(+)-Oxyglutinosone	$C_{14}H_{20}O_3$	1.45E+06	0.04
289.17421	Aristolasene	$C_{20}H_{22}N_2$	1.12E+06	0.03
279.13467	(+)-conocarpan methyl ether|(2S,3S)-2,3-dihydro-2-(4-methoxyphenyl)-3-methyl-5-[1(E)-propenyl]benzofuran	$C_{19}H_{20}O_2$	5.04E+06	0.13
511.27271	(+/-) pseudophrynamine A|Pseudophrynamine A	$C_{32}H_{40}N_4O_2$	1.93E+06	0.05
245.09229	(-)-(1R,3S)-7-hydroxy-1-methyl-2,3,4,9-tetrahydro-1H-beta-carboline-3-carboxylic acid|brunnein B	$C_{13}H_{14}N_2O_3$	2.13E+06	0.05
247.16921	(1S,5S,9R)-5-methoxycaryophylla-2(15),6(14)-dien-7-one	$C_{16}H_{24}O_2$	4.95E+06	0.12
243.10257	(1S,8aS)-1-(3-furyl)-5,8a-dimethyl-7,8-dihydro-1H-isochromen-3-one	$C_{15}H_{16}O_3$	1.96E+06	0.05
263.11115	(1Z,3E)-Chlorohexadeca-1,3-diene-5,7-diyne-14-ol	$C_{16}H_{21}ClO$	2.13E+06	0.05
277.18255	(2E)-3-formyl-5-(2',6',6'-trimethylcyclohex-2'-enyl)pent-2-enyl acetate	$C_{17}H_{26}O_3$	2.21E+06	0.06
293.21767	(2R)-sahandol	$C_{21}H_{26}O$	2.88E+06	0.07
251.1273	(2R,5R,8S,8aR)-1,2,3,5,6,7,8,8a-octahydro-5-hydroxy-8,8a-dimethyl-3-oxonaphthalene-2-yl acetate	$C_{14}H_{20}O_4$	1.04E+06	0.03
243.0993	(2S,4S)-Pinnatanine	$C_{10}H_{16}N_2O_5$	1.49E+06	0.04
279.19897	(3E,7E)-6-hydroxy-9-isopropyl-6-methyltrideca-3,7-diene-2,12-dione|6-Hydroxy-9-ispropyl-6-methyl-3,7-tridecadiene-2,12-dione	$C_{17}H_{28}O_3$	1.59E+06	0.04
295.19395	(3S,6S)-3-acetoxy-2,2,6-trimethyl-6-([1S,4R]-4-hydroxy-4-methylcyclohex-2-en-1-yl)tetrahydropyran	$C_{17}H_{28}O_4$	1.64E+06	0.04
279.13235	(4E)-6-hydroxy-1,7-diphenylhept-4-en-3-one	$C_{19}H_{20}O_2$	5.52E+06	0.14
397.37166	(4S,6S)-2,3-erythro-3-Hydroxy-4,6-dimethyl-heneicosancarbonsaeure-(2)-methylester; Mycolipanolsaeuremethylester	$C_{25}H_{50}O_3$	1.29E+06	0.03
123.06048	(5-methylpyrazin-2-yl)methanol	$C_6H_8N_2O$	1.29E+06	0.03
281.14467	(5E)-2-ethylimino-5-(1H-indol-3-ylmethylidene)-1,3-dimethylimizolidin-4-one	$C_{16}H_{18}N_4O$	5.02E+06	0.13

279.23251	(6E,10E)-14-hydroxy-2,6,10-trimethyl-pentadeca-6,10-dien-4-one	$C_{18}H_{32}O_2$	2.43E+07	0.61
279.24157	(6E,11Z)-6,11-Octadecadienoic acid|6,11-Octadecadienoic acid	$C_{18}H_{32}O_2$	1.70E+07	0.43
217.12252	(6S)-4-hydroxy-14-norcadina-1,3,5-triene-9-one|oxyphyllone G	$C_{14}H_{18}O_2$	1.12E+06	0.03
291.19541	(9S,10E,16R)-octadec-10-ene-12,14-diyne-1,9-16-triol	$C_{18}H_{28}O_3$	2.48E+06	0.06
261.13366	(E)-2-Hexenyl Beta-D-Glucopyranoside|(E)-2-hexenyl beta-D-glucoside|(E)-2-hexenyl-beta-D-glucopyranoside|(E)-2-Hexenyl-??-D-glucopyranoside|erigeside B	$C_{12}H_{22}O_6$	1.03E+06	0.03
261.10935	(E)-[5-(5-Methylhtien-2-yl)-2-penten-4-ynyl]-3-methylbutanoate	$C_{15}H_{18}O_2S$	1.28E+07	0.32
281.24658	(E,R)-6,7-dimethylhexadec-7-enoic acid	$C_{18}H_{34}O_2$	1.71E+07	0.43
297.24302	(R)-11-Cycloheptyl-2-hydroxyundecanoic acid	$C_{18}H_{34}O_3$	1.75E+06	0.04
97.05753	(S)-1-Hexyn-3-ol|1-Hexyn-3-ol	$C_6H_{10}O$	1.02E+06	0.03
291.18095	(S)-3-Octanol glucoside	$C_{14}H_{28}O_6$	1.24E+06	0.03
245.04922	(S)-4-(4-hydroxyphenyl)-2-butanol 2-O-sulfate	$C_{10}H_{14}O_5S$	1.87E+06	0.05
235.13294	(S)-dihydrodemethoxywutaiensol|3-[(2S)-2,3-dihydro-2-(2-hydroxypropan-2-yl)-1-benzofuran-5-yl]propan-1-ol	$C_{14}H_{20}O_3$	1.26E+06	0.03
245.19236	(S)-panaxjapyne A|(Z)-heptadec-9-ene-4,6-diyn-(3S)-ol|panaxjapyne A	$C_{17}H_{26}O$	2.47E+06	0.06
261.13538	(Z)-3-Hexen-1-yl b-D-glucopyranoside	$C_{12}H_{22}O_6$	1.37E+06	0.03
279.12173	(Z,Z)-1,12-Diisothiocyanato-1,11-dodecadiene	$C_{14}H_{20}N_2S_2$	6.64E+06	0.17
245.0457	1'-Benzoyl-3-Hydroxy-2-(hydroxymethyl)-4H-Pyran-4-one	$C_{13}H_{10}O_5$	1.08E+06	0.03
243.12371	1,2,3,4-Di-O-isopropylidene-a-D-fucopyranose	$C_{12}H_{20}O_5$	1.08E+06	0.03
191.05788	1,3-Dimethyllumazine	$C_8H_8N_4O_2$	2.19E+06	0.06
277.11773	1,4-Dimethyl-2-hydroxy-2-(4-methoxybenzyl)piperazine-3,6-dione	$C_{14}H_{18}N_2O_4$	1.25E+06	0.03
261.09153	1,6-dimethylpyrene-2,7-diol	$C_{18}H_{14}O_2$	1.60E+06	0.04
217.12536	1,8-Dimethyl-3a-methoxy-1,2,3,3a,8,8a-hexahydropyrrolo[2,3-b]indole	$C_{13}H_{18}N_2O$	4.50E+06	0.11
311.298	1-(2-hydroxyethyl)-2-(12-aminotridecyl)pyrrolidine	$C_{19}H_{40}N_2O$	2.05E+06	0.05
249.14597	1-DODECANESULFONIC ACID	$C_{12}H_{26}O_3S$	2.06E+07	0.52
353.34522	1-Tetracosanol	$C_{24}H_{50}O$	2.98E+06	0.08
301.18284	1-[(4aS,10aR)-1,2,3,4,4a,9,10,10a-octahydro-8-hydroxy-1-(hydroxymethyl)-1,4a-dimethylphenanthren-7-yl]ethanone|13-acetyl-14,15-dihydroxypodocarpa-8,11,13-triene	$C_{19}H_{26}O_3$	1.19E+06	0.03

219.10462	1-benzylquinolinium	$C_{16}H_{14}N$	1.26E+06	0.03
285.05283	1-methanol-5-[4-(2-propen-1-yl)phenoxy]-2,3,4-benzenetriol|2-hydroxyobovaaldehyde	$C_{16}H_{14}O_5$	4.34E+06	0.11
229.14658	10-ACETOXYDECANOIC ACID	$C_{12}H_{22}O_4$	1.42E+06	0.04
297.24908	10-Oxooctadecanoic acid	$C_{18}H_{34}O_3$	1.48E+06	0.04
245.16481	11,12-Dehydrolupanine	$C_{15}H_{22}N_2O$	2.00E+06	0.05
333.20971	11alpha,12beta-epoxy-5alpha-hydroxy-1betaH,2alphaH-casba-3Z,7E-dien-18-oic acid|pekinenin E	$C_{20}H_{30}O_4$	1.77E+06	0.04
246.128035	12-Cytisineacetamide	$C_{13}H_{17}N_3O_2$	5.84E+07	1.47
295.22562	12-hydroxyoctadeca-9,13-dienoic acid	$C_{18}H_{32}O_3$	3.86E+06	0.10
301.21499	12beta-hydroxy-8(9),15-isopimaradien-7-one	$C_{20}H_{30}O_2$	9.83E+06	0.25
217.12375	13-desoxyonoseriolide	$C_{14}H_{18}O_2$	1.91E+06	0.05
277.19142	13alpha-Methoxylupanine	$C_{16}H_{26}N_2O_2$	2.35E+06	0.06
291.16733	14-Acetoxy-11alpha,13-dihydrodesoxyivangustin	$C_{17}H_{24}O_4$	1.29E+07	0.33
299.26664	14-hydroxy stearic acid	$C_{18}H_{36}O_3$	1.88E+06	0.05
253.21706	14-methyl-6-pentadecenoic acid	$C_{16}H_{30}O_2$	1.00E+06	0.03
255.26588	14-methylhexadecan-1-ol	$C_{17}H_{36}O$	1.06E+06	0.03
279.25611	15-Nor-8-labnol	$C_{19}H_{36}O$	2.54E+06	0.06
341.11587	15-O-acetyl-3-deoxy-11alpha-hydroxy-13-chloro-11,13-dihydroamphoricarpolide	$C_{17}H_{23}ClO_5$	1.31E+06	0.03
297.24061	16-hydroxyhexadec-9-en-7-yl acetate	$C_{18}H_{34}O_3$	1.33E+06	0.03
279.23985	17-Octadecynoic acid	$C_{18}H_{32}O_2$	1.97E+07	0.50
299.25034	18-hydroxyoctadecanoic acid	$C_{18}H_{36}O_3$	1.06E+06	0.03
82.043715	1H-Imizol-2-amine	$C_3H_5N_3$	3.62E+06	0.09
281.13451	1alpha-Hydroxyalliacolide	$C_{15}H_{22}O_5$	3.92E+06	0.10
247.09697	2,2,5-Trimethyl-7-hydroxy-2H-1-benzopyran-6-carboxylic acid methyl ester	$C_{14}H_{16}O_4$	1.57E+06	0.04
285.04949	2,3,4-trihydroxy-4'-methoxychalcone	$C_{16}H_{14}O_5$	5.64E+06	0.14
297.14016	2,4,5-Triphenyl-2-imizoline	$C_{21}H_{18}N_2$	1.08E+06	0.03
255.21231	2,4b-Dimethyl-8-methylene-2-vinyl-1,2,3,4,4a,4b,5,6,7,8,8a,9-dodecahydrophenanthrene #	$C_{19}H_{28}$	1.81E+06	0.05

241.22136	2,6,8-Trimethyl-undecansaeure-methylester	$C_{15}H_{30}O_2$	1.04E+06	0.03
233.1204	2,6-Diamino-7-hydroxy-azelaic acid	$C_9H_{18}N_2O_5$	6.37E+06	0.16
297.12889	2-(1-Methyl-1H-pyrrol-2-yl)-3-(3,4,5-trimethoxyphenyl)acrylonitrile	$C_{17}H_{18}N_2O_3$	1.09E+06	0.03
285.04288	2-(2,4-dihydroxyphenyl)-3,7-dihydroxychromen-4-one	$C_{15}H_{10}O_6$	1.94E+07	0.49
97.05659	2-CYCLOHEXEN-1-OL	$C_6H_{10}O$	1.32E+06	0.03
285.01877	2-Chloro-1,3,5-benzenetriol triacetate	$C_{12}H_{11}ClO_6$	1.07E+06	0.03
250.16221	2-Diphenylmethylpiperidine	$C_{18}H_{21}N$	2.77E+06	0.07
89.05593	2-Ethoxyethanol	$C_4H_{10}O_2$	2.13E+06	0.05
281.25726	2-Hexadecenoic acid, ethyl ester, (2E)-	$C_{18}H_{34}O_2$	1.42E+07	0.36
249.15849	2-Isopropyl-4-methoxy-5-methylphenyl isobutyrate	$C_{15}H_{22}O_3$	1.62E+07	0.41
245.09938	2-Phenoxybiphenyl	$C_{18}H_{14}O$	1.20E+07	0.30
201.12889	2-Prenyl-4-allylphenol	$C_{14}H_{18}O$	3.82E+06	0.10
247.16668	2-benzyloxy-5-pentyltetrahydrofuran	$C_{16}H_{24}O_2$	5.96E+06	0.15
241.21595	2-heptyl octanoate	$C_{15}H_{30}O_2$	1.00E+06	0.03
285.03287	2-methoxy-6-[5-(prop-1-yn-1-yl)-2-thienyl]-hexa-3,5-diyn-1-yl acetate	$C_{16}H_{14}O_3S$	3.51E+06	0.09
83.05197	2-methylbut-2-enal	C_5H_8O	2.49E+06	0.06
83.05241	2-methylcyclobutan-1-one	C_5H_8O	2.47E+06	0.06
283.26386	2-nonyloxy-5-pentyltetrahydrofuran	$C_{18}H_{36}O_2$	9.54E+06	0.24
277.247	2-pentadecylfuran	$C_{19}H_{34}O$	1.09E+06	0.03
355.32451	20-Methyl-docosandiol-(1,2)|20-methyl-docosane-1,2-diol	$C_{23}H_{48}O_2$	4.65E+06	0.12
367.35608	21-methyltricosanoic acid	$C_{24}H_{48}O_2$	1.30E+06	0.03
255.26411	3,7-dimethylpentadecan-2-ol	$C_{17}H_{36}O$	1.08E+06	0.03
889.08013	3-(3,5-diiodo-4-methoxyphenyl)-3'-(3-iodo-4-methoxyphenyl)-N,N'-(pentane-1,5-diyl)bis(2-dimethylaminopropanamide)|N29-Me-Alpha-(Dimethylamino)-3,5-diiodo-N-[5-[[3-(3-iodo-4-methoxyphenyl)-2-(methylamino)-1-oxopropyl]amino]pentyl]-4-methoxybenzenepropanamide	$C_{29}H_{41}I_3N_4O_4$	1.51E+06	0.04
262.12433	3-(p-fluorobenzoyloxy)tropane	$C_{15}H_{18}FNO_2$	1.59E+06	0.04
186.10674	3-amino-beta-pinene	$C_{10}H_{18}ClN$	1.91E+06	0.05
82.04266	3-Aminopyrazole	$C_3H_5N_3$	2.09E+07	0.53

85.08041	3-Aminopyrrolidine	$C_4H_{10}N_2$	1.17E+07	0.29
245.1368	3-Thiatetradecanoic Acid	$C_{13}H_{26}O_2S$	2.04E+07	0.52
243.10831	3-hydroxy-5-guanidino-2-(2-oxoazetidin-1-yl)pentanoic acid	$C_9H_{16}N_4O_4$	1.54E+06	0.04
291.15188	3-oxodecan-1-ol p-hydroxybenzoate	$C_{17}H_{24}O_4$	7.09E+06	0.18
248.14538	4'-Fluoro-alpha-pyrrolidinopentiophenone	$C_{15}H_{20}FNO$	1.95E+07	0.49
246.13029	4,6-Dideoxy-3-C-methyl-4-(methuylamino)mannose,9CI-beta-D-Pyranose-form-Me glycoside,N-Ac	$C_{11}H_{21}NO_5$	2.13E+07	0.54
248.12841	4-Coumaroylcholine	$C_{14}H_{19}NO_3$	4.47E+06	0.11
325.18339	4-Dodecylbenzenesulfonic acid	$C_{18}H_{30}O_3S$	1.18E+06	0.03
295.23506	4-Oxooctadec-2-enoic acid	$C_{18}H_{32}O_3$	3.66E+06	0.09
245.24706	4-Phenyldodecane	$C_{18}H_{30}$	1.48E+06	0.04
267.17125	4-[1-Ethyl-2-(4-methylphenyl)butyl]phenol	$C_{19}H_{24}O$	4.41E+06	0.11
233.16001	4-[4-(4-hydroxybutoxy)butoxy]butan-1-ol	$C_{12}H_{26}O_4$	3.25E+06	0.08
509.20427	4-[[4'-hydroxy-4-(hydroxymethyl)-3-[2-(4-hydroxyphenyl)ethynyl][1,1'-biphenyl]-2-yl](4-methoxyphenyl)methylene]-2,5-cyclohexadien-1-one/selaginellin M	$C_{35}H_{26}O_4$	1.41E+06	0.04
82.0431225	4-aminoimizole	$C_3H_5N_3$	9.95E+06	0.25
271.23976	5,15-Rosadiene	$C_{20}H_{32}$	2.17E+06	0.05
295.23031	5-(3-Methoxycarbonylbutyroyl)aminomethyl-cis-quinolizidine	$C_{16}H_{28}N_2O_3$	9.65E+06	0.24
285.0285	5-(4-chlorophenyl)-3-phenyl-1H-imizole-2-thione	$C_{15}H_{11}ClN_2S$	1.76E+06	0.04
245.03662	5-Chloro-2-hydroxy-4-methylbenzophenone	$C_{14}H_{11}ClO_2$	1.09E+06	0.03
247.10218	5-Ethenyl-1,6-dimethylphenanthrene-2-ol	$C_{18}H_{16}O$	2.66E+06	0.07
279.23097	5-Dodecanyl-4-hydroxy-4-meth-yl-2-cyclopentenone	$C_{18}H_{32}O_2$	2.03E+07	0.51
255.25255	6,10,13-trimethyltetradecan-1-ol	$C_{17}H_{36}O$	2.57E+06	0.06
509.27263	6-Hydroxy-Thionuphlutine B	$C_{30}H_{42}N_2O_3S$	5.06E+06	0.13
299.054665	6-chloro-1-pentofuranosyl-1h-imizo[4,5-c]pyridin-4-amine	$C_{11}H_{13}ClN_4O_4$	2.72E+06	0.07
305.15454	6-methyl-8-prenylflavanone	$C_{21}H_{22}O_2$	1.07E+06	0.03

235.134	6alpha-acetyl-4beta,5beta-dimethyl-1(10)-ene-2alpha-hydroxy-7-oxodecalin|laevinol E	$C_{14}H_{20}O_3$	1.33E+06	0.03
285.04004	7,8,2',4'-Tetrahydroxyisoflavone	$C_{15}H_{10}O_6$	7.72E+06	0.19
262.115	7-Mercaptoheptanoylthreonine	$C_{11}H_{21}NO_4S$	1.78E+06	0.04
279.12336	7-carboxyl-6,8-dihydroxy-1,1,3alpha,4beta,5-pentamethylisochroman	$C_{15}H_{20}O_5$	8.00E+06	0.20
509.21949	7beta-acetyl-6beta-benzoyl-12,16-epoxy-5alpha-hydroxy-12alpha-methoxycassa-12,15-dien-19beta-oic acid|pulcherrimin F	$C_{29}H_{34}O_8$	2.26E+06	0.06
277.20603	8-(2,2-Dimethyl-3-hydroxy-6-methylenecyclobexyl)-6-methyl-5-octen-2-one|8-(5-Hydroxy-2,6,6-trimethyl-1-cyclohexenyl)-6-methyl-5-octen-2-one	$C_{18}H_{30}O_2$	2.02E+06	0.05
245.08162	8-(2-Thienyl)-3t,5t-octadien-7-in-1-ol-acetat|Ac-(3E,5E)-8-(2-Thienyl)-3,5-octadien-7-yl-1-ol	$C_{14}H_{14}O_2S$	3.08E+06	0.08
291.19177	8-[3-oxo-2-[(E)-pent-2-enyl]cyclopenten-1-yl]octanoic acid	$C_{18}H_{28}O_3$	2.77E+06	0.07
269.21714	8-oxohexadecanoic acid	$C_{16}H_{30}O_3$	1.38E+06	0.03
329.23988	9,10-Dihydroxy-12,13-epoxyoctadecanoate	$C_{18}H_{34}O_5$	1.11E+06	0.03
341.10513	9-[(Methoxymethyl)amino]-8H-benzo[b]pyrido[4,3,2-de][1,10]phenanthroline-8-one	$C_{20}H_{14}N_4O_2$	1.15E+06	0.03
251.17246	9-hydroxyfarnesoic acid	$C_{15}H_{24}O_3$	5.12E+06	0.13
281.25383	Ac-(E)-7-Hexadecen-1-ol|Hexalure	$C_{18}H_{34}O_2$	2.01E+07	0.51
291.21281	Aikupikanyne C	$C_{22}H_{28}$	1.51E+06	0.04
254.12581	Aleph-2	$C_{13}H_{21}NO_2S$	4.61E+06	0.12
245.1633	Aphyllidine	$C_{15}H_{22}N_2O$	1.05E+06	0.03
295.23339	Aplysillamide A	$C_{16}H_{32}N_4O$	4.39E+06	0.11
250.16419	Arglecin	$C_{12}H_{21}N_5O$	2.00E+06	0.05
555.28817	Artocommunol CB	$C_{35}H_{40}O_6$	2.48E+06	0.06
289.1318	Benomyl	$C_{14}H_{18}N_4O_3$	2.00E+06	0.05
532.24396	Benzamide, 4-amino-N-[1-[2,6-dideoxy-4-O-[4,6-dideoxy-4-(dimethylamino)-alpha-D-glucopyranosyl]-beta-D-arabino-hexopyranosyl]-1,2-dihydro-2-oxo-4-pyrimidinyl]-	$C_{25}H_{35}N_5O_8$	1.04E+06	0.03
301.19245	Benzastatin I	$C_{18}H_{26}N_2O_2$	1.83E+06	0.05

385.29871	Bourgeanic-Acid	$C_{22}H_{42}O_5$	1.07E+06	0.03
297.13252	Brasiloide	$C_{15}H_{22}O_6$	1.34E+06	0.03
383.34774	Buxamin E	$C_{26}H_{44}N_2$	1.22E+06	0.03
97.05702	Cyclohexanone	$C_6H_{10}O$	1.28E+06	0.03
83.05176	Cyclopentanone	C_5H_8O	1.84E+06	0.05
233.16494	Camoensidine	$C_{14}H_{22}N_2O$	1.69E+06	0.04
254.1199	Carbazomycin A	$C_{16}H_{17}NO_2$	1.10E+06	0.03
555.2546	Chaetoglobosin L	$C_{34}H_{40}N_2O_5$	1.50E+06	0.04
285.03559	Cinnabarine	$C_{14}H_{10}N_2O_5$	5.36E+06	0.14
254.13108	Clobutinol	$C_{14}H_{22}ClNO$	1.23E+06	0.03
325.18098	Cordiaquinone A	$C_{21}H_{26}O_3$	1.03E+06	0.03
279.268865	Cyclononadecanone	$C_{19}H_{36}O$	2.38E+06	0.06
245.12049	DL-Hypaphorine	$C_{14}H_{18}N_2O_2$	1.01E+09	25.40
245.16597	Dehydrovirgiboidine	$C_{15}H_{22}N_2O$	2.53E+06	0.06
247.2198	Dihydrodeoxycernuine	$C_{16}H_{28}N_2$	1.70E+06	0.04
262.12626	Dimepiperate	$C_{15}H_{21}NOS$	1.44E+06	0.04
277.18933	Dimethocaine	$C_{16}H_{26}N_2O_2$	2.85E+06	0.07
93.04395	Dinitrile-Pentanedioic acid	$C_5H_6N_2$	2.38E+06	0.06
317.21471	Dioxime-5alpha-Androstane-3,17-dione	$C_{19}H_{30}N_2O_2$	2.39E+06	0.06
262.12132	Dioxypyramidon	$C_{13}H_{17}N_3O_3$	2.00E+06	0.05
247.13075	Diptocarpamine	$C_{11}H_{24}N_2O_2S$	9.28E+07	2.34
362.20002	Euchrestine B	$C_{24}H_{29}NO_2$	2.19E+06	0.06
95.01626	Furfural	$C_5H_4O_2$	1.75E+06	0.04
302.23486	Fenpropimorph	$C_{20}H_{33}NO$	1.21E+06	0.03
311.18312	Ficellomycin	$C_{13}H_{24}N_6O_3$	1.11E+06	0.03
245.14041	Fulvoferruginin	$C_{15}H_{18}O_3$	1.72E+07	0.43
325.17729	Furospongin 5	$C_{21}H_{26}O_3$	1.36E+06	0.03

571.295	Ganoderic acid H	$C_{32}H_{44}O_9$	1.01E+06	0.03
247.09164	Glypetelotine	$C_{13}H_{16}N_2OS$	1.90E+06	0.05
251.16741	Hexazinone	$C_{12}H_{20}N_4O_2$	2.32E+07	0.59
235.13091	Halimedin	$C_{10}H_{16}N_6O$	1.09E+06	0.03
267.15602	Histidinyl-Isoleucine	$C_{12}H_{20}N_4O_3$	5.67E+06	0.14
277.22594	Huperzine J	$C_{17}H_{30}N_2O$	6.34E+06	0.16
267.15349	Isomethiozin	$C_{12}H_{20}N_4OS$	3.33E+06	0.08
889.50235	Kulokainalide-1	$C_{48}H_{70}N_6O_{10}$	1.60E+06	0.04
265.16732	L-Sorbitol permethyl	$C_{12}H_{26}O_6$	1.62E+06	0.04
285.04572	Leptanthin	$C_{12}H_{18}N_2O_2S_2$	7.64E+06	0.19
245.12403	Ligudentatin A	$C_{15}H_{18}O_3$	2.12E+08	5.36
243.11232	Limazepine F	$C_{14}H_{16}N_2O_2$	1.43E+06	0.04
246.09448	Linamarin	$C_{10}H_{17}NO_6$	1.49E+06	0.04
271.233685	Louludinium	$C_{19}H_{30}N$	6.96E+06	0.18
97.05682	Mesityl oxide	$C_6H_{10}O$	1.32E+06	0.03
245.17951	Monocaprin	$C_{13}H_{26}O_4$	1.85E+06	0.05
247.0939	Malioxamycin	$C_9H_{16}N_2O_6$	1.33E+06	0.03
692.31422	Manshuritine	$C_{38}H_{47}NO_{11}$	2.62E+06	0.07
247.17714	Matrin	$C_{15}H_{24}N_2O$	1.10E+06	0.03
255.24924	Methyl 13-methyltetradecanoate	$C_{16}H_{32}O_2$	2.41E+06	0.06
367.35284	Methyl tricosanoate	$C_{24}H_{48}O_2$	1.07E+06	0.03
299.26165	Methyl-7-methoxypalmitat	$C_{18}H_{36}O_3$	6.32E+06	0.16
245.16824	Myristoyl chloride	$C_{14}H_{27}ClO$	2.98E+06	0.08
256.22889	N-(2-Hydroxypropyl)dodecanamide	$C_{15}H_{31}NO_2$	1.76E+06	0.04
246.09955	N-(3-imizol-1-ylpropyl)-3-nitropyridin-2-amine	$C_{11}H_{13}N_5O_2$	2.13E+06	0.05

289.13954	N-(4-Chlor-benzyl)-N',N'-dimethyl-N-pyrimidin-2-yl-aethylendiamin丨N-(4-chloro-benzyl)-N',N'-dimethyl-N-pyrimidin-2-yl-ethylenediamine丨solamin	$C_{15}H_{19}ClN_4$	6.08E+06	0.15
543.2632	NSC709845	$C_{30}H_{36}N_6O_4$	2.76E+06	0.07
247.15259	Narbosine D	$C_{12}H_{24}O_5$	1.58E+07	0.40
525.25273	Nupharpumilamine B	$C_{30}H_{42}N_2O_4S$	4.69E+06	0.12
509.24775	Nupharpumilamine D	$C_{30}H_{42}N_2O_3S$	4.74E+06	0.12
291.15968	O1-(1-isopropyl-2-methyl-propyl)-beta-D-glucopyranuronic acid丨O1-(1-Isopropyl-2-methyl-propyl)-beta-D-glucopyranuronsaeure	$C_{13}H_{24}O_7$	1.62E+07	0.41
283.27311	Octadec-9-ene-1,18-diol	$C_{18}H_{36}O_2$	6.08E+06	0.15
83.05271	Pent-3-enal	C_5H_8O	2.14E+06	0.05
247.16096	Phenethyl octanoate	$C_{16}H_{24}O_2$	9.65E+06	0.24
246.15399	Phlegmariurine B	$C_{15}H_{21}NO_2$	1.94E+06	0.05
239.16738	Phomapentenone A	$C_{14}H_{24}O_3$	1.61E+06	0.04
266.15906	Pipradrol	$C_{18}H_{21}NO$	1.28E+06	0.03
245.107017	Prolyl-Methionine	$C_{10}H_{18}N_2O_3S$	1.78E+08	4.50
218.02789	Pulicatin D	$C_{11}H_9NO_2S$	1.26E+06	0.03
555.26239	Roridin E acetate	$C_{31}H_{40}O_9$	1.16E+06	0.03
301.22952	Stearoyl chloride	$C_{18}H_{35}ClO$	7.24E+06	0.18
249.17204	Scortechterpene B	$C_{16}H_{26}O_2$	3.21E+06	0.08
285.03835	Scutellarein	$C_{15}H_{10}O_6$	6.25E+06	0.16
291.18873	Sorelline	$C_{20}H_{24}N_2$	2.25E+06	0.06
284.27903	Spisulosine; Spisulosine 285	$C_{18}H_{39}NO$	1.33E+06	0.03
277.19593	Tetradecylphosphonic acid	$C_{14}H_{31}O_3P$	4.68E+06	0.12
245.05977	Thiostreptine	$C_9H_{14}N_2O_4S$	1.88E+06	0.05
277.15599	Tolycaine	$C_{15}H_{22}N_2O_3$	1.39E+06	0.04

313.13171	Triazamate	$C_{13}H_{22}N_4O_3S$	1.00E+06	0.03
247.08814	Tuboflavine	$C_{16}H_{12}N_2O$	2.15E+06	0.05
293.21459	[(3aR,6aR,7S,9aS,9bR)-3a,4,6a,7,8,9,9a,9b-octahydro-2,2,9a-trimethyl-7-(1-methylethyl)azuleno[4,5-d][1,3]dioxol-5-yl]methanol	$C_{18}H_{30}O_3$	9.87E+06	0.25
335.22534	Acebutolol	$C_{18}H_{28}N_2O_4$	3.73E+06	0.09
509.2425	Astrogorgin G	$C_{26}H_{38}O_{10}$	3.90E+06	0.10
427.20087	Benzodioxolefentanyl	$C_{27}H_{28}N_2O_3$	1.15E+06	0.03
245.06496	bis-(4-hydroxybenzyl)sulfide	$C_{14}H_{14}O_2S$	2.58E+06	0.06
227.20368	Butan-2-yl decanoate	$C_{14}H_{28}O_2$	2.10E+06	0.05
254.128825	cis-Nalphacinnamoyl-N1-methylhistamine	$C_{15}H_{17}N_3O$	2.78E+06	0.07
355.31292	Conessimine	$C_{24}H_{40}N_2$	1.19E+06	0.03
247.16343	Dihydro-epideoxyarteannuin B	$C_{16}H_{24}O_2$	5.54E+06	0.14
201.12496	DL-Alanyl-DL-Isoleucine	$C_9H_{18}N_2O_3$	1.23E+06	0.03
233.15155	Erectathiol|[(6R,9S)-6-isopropyl-9-methyl-6,7,8,9-tetrahydronaphthalen-3-yl]methanethiol	$C_{15}H_{22}S$	3.19E+06	0.08
97.05626	Hex-3-enal	$C_6H_{10}O$	3.48E+06	0.09
334.16879	Hydroxychloroquine	$C_{18}H_{26}ClN_3O$	1.48E+06	0.04
261.10701	Isoleucylmethionine	$C_{11}H_{22}N_2O_3S$	8.22E+06	0.21
261.12738	Methionylisoleucine	$C_{11}H_{22}N_2O_3S$	4.64E+06	0.12
349.20474	Methyl 4beta-hydroxy-6-oxo-19-norgrindeloate	$C_{20}H_{30}O_5$	1.10E+06	0.03
291.16932	Normelanothyrsin A	$C_{17}H_{24}O_4$	1.13E+07	0.29
250.14724	o-Methylpellotine	$C_{14}H_{21}NO_3$	2.19E+06	0.06
281.24821	Octadec-16-enoic acid	$C_{18}H_{34}O_2$	2.01E+07	0.51
281.24362	Octadec-8-enoic acid	$C_{18}H_{34}O_2$	1.43E+07	0.36
281.2594	Oxacyclononadecan-2-one	$C_{18}H_{34}O_2$	1.20E+07	0.30

341.10095	p-Nitrophenyl-N-acetyl-beta-D-glucosaminide	$C_{14}H_{18}N_2O_8$	1.29E+06	0.03
83.05148	Pent-3-en-2-one	C_5H_8O	1.88E+06	0.05
283.16194	Phomoenamide	$C_{14}H_{24}N_2O_4$	2.25E+06	0.06
387.11712	Propionic acid 4 sodium	$C_{12}H_{24}Na_4O_8$	1.26E+06	0.03
289.14404	Sarcobiose	$C_{13}H_{22}O_7$	9.13E+06	0.23
509.23434	Scutiaquinone B	$C_{32}H_{30}O_6$	9.44E+06	0.24
289.14991	Serylglycyllysine	$C_{11}H_{22}N_4O_5$	8.37E+06	0.21
245.06833	Spongiacidin C	$C_{11}H_{10}N_4O_3$	2.02E+06	0.05
217.12699	Tetradeca-5,7,9,11,13-pentaenoic acid	$C_{14}H_{18}O_2$	1.77E+06	0.04
889.52732	Tetrahydrogeranylgeranyl chlorophyll a	$C_{55}H_{70}MgN_4O_5$	1.18E+06	0.03
283.25934	Totarol	$C_{21}H_{32}$	6.59E+06	0.17
305.21295	Valeric acid 3	$C_{15}H_{30}O_6$	1.00E+06	0.03

* All metabolite annotations are putative and were assigned based on accurate mass and database matching. Structural confirmation requires MS/MS fragmentation and/or authentic standards.

$^{\alpha}$Total sum of peak abundance and % relative abundance of these compounds in the negative ESI mode.

Table S3. Phytochemicals peaks identified in MB-LE via FI-ESI-FT-ICR MS analysis in negative electrospray ionization mode (ESI-, ≤20 ppm).

m/z	Putative metabolite annotation*	Molecular Formula	Peak Abundance[α]	% Relative Abundance[α]
383.34652	(+)-Buxamine F	$C_{26}H_{44}N_2$	3.20E+06	0.08
249.15174	(3S,6S)-3-((1H-imizol-4-yl)methyl)-6-isobutylpiperazine-2,5-dione∣cis-cyclo-(His-Leu)∣cyclo-(L-leucyl-L-histidyl)	$C_{12}H_{18}N_4O_2$	1.21E+08	3.05
123.06455	1-(1H-PYRROL-2-YL)Ethan-1-oneoxime	$C_6H_8N_2O$	8.28E+06	0.21
247.14682	1-chlorotridec-1-ene-6,8-diol	$C_{13}H_{25}ClO_2$	3.28E+07	0.83
279.22807	10,12-hexadecadienyl acetate	$C_{18}H_{32}O_2$	1.87E+07	0.47
295.123005	12, 13-Dihydro, Ac-(3E, 6S, 7S, 9Z, 12Z)-7-Chloro-3, 9, 12-pentadecatrien-1-yn-6-ol	$C_{17}H_{25}ClO_2$	4.25E+06	0.11
255.23894	13-methylpentadecanoic acid	$C_{16}H_{32}O_2$	2.27E+07	0.57
124.070435	2-Acetyl-1,4,5,6-tetrahydropyridine	$C_7H_{11}NO$	6.46E+06	0.16
255.228552	2-N-Dodecyltetrahydrothiophene	$C_{16}H_{32}S$	1.45E+08	3.66
245.10762	2-[(2'-methylsulfanyl)hexyl]maleate	$C_{11}H_{18}O_4S$	2.45E+07	0.62
555.32494	3beta-(benzoyloxy)glutina-5(10),6-dien-27,8alpha-olide∣phyllenolide B	$C_{37}H_{48}O_4$	4.11E+06	0.10
311.22369	4,6-Diethyl-6-(2-methylhexyl)-3,6-dihydro-1,2-dioxin-3-acetic acid methyl ester	$C_{18}H_{32}O_4$	3.68E+06	0.09
123.062815	4,6-Dimethyl-2-hydroxypyrimidine	$C_6H_8N_2O$	4.67E+06	0.12
246.10522	4,6-diphenylpyrimidin-2-amine	$C_{16}H_{13}N_3$	9.28E+06	0.23
283.2796	4-Methyloctadecan-1-ol	$C_{19}H_{40}O$	7.06E+06	0.18
255.25582	9-Heptadecanol	$C_{17}H_{36}O$	2.68E+06	0.07
201.12799	Dictagymnin	$C_{14}H_{18}O$	2.65E+06	0.07
293.222915	Diphenyldecane	$C_{22}H_{30}$	4.33E+06	0.11
255.260045	Heptadecan-1-ol	$C_{17}H_{36}O$	3.50E+06	0.09
283.26668	Hexadecyl acetate	$C_{18}H_{36}O_2$	2.36E+07	0.59
255.24037	Methyl 2,8-dimethyltridecanoate	$C_{16}H_{32}O_2$	1.99E+07	0.50

279.23532	Methyl aphyllate	$C_{16}H_{28}N_2O_2$	7.83E+07	1.98
123.062777	N-Propanoylimizole	$C_6H_8N_2O$	1.35E+07	0.34
246.13703	N1-(1,3,5-Trimethyl-1H-pyrazol-4-yl)-2-cyano-3-(dimethylamino)acrylamide	$C_{12}H_{17}N_5O$	3.71E+06	0.09
282.264805	Octadecanamide	$C_{18}H_{37}NO$	3.27E+06	0.08
377.081427	Pyrite	$C_{19}H_{23}ClN_2O_2S$	2.43E+07	0.61
201.133727	Symmetric dimethylarginine	$C_8H_{18}N_4O_2$	7.36E+06	0.19
509.21861	Tubastrindole H	$C_{28}H_{26}N_6O_4$	5.91E+06	0.15
245.079263	Brussalexin A	$C_{13}H_{14}N_2OS$	1.12E+07	0.28
87.0403643	Butyric acid	$C_4H_8O_2$	1.32E+07	0.33
301.20616	Emestine	$C_{17}H_{26}N_4O$	7.58E+06	0.19
85.0797325	Piperazine	$C_4H_{10}N_2$	8.55E+06	0.22
295.122875	Propionic acid 4	$C_{12}H_{24}O_8$	2.43E+06	0.06
249.122745	Valeric acid 2 sodium	$C_{10}H_{20}Na_2O_4$	2.90E+06	0.07
246.14838	Mioporosidegenin	$C_{12}H_{22}O_5$	2.67E+06	0.07

* All metabolite annotations are putative and were assigned based on accurate mass and database matching. Structural confirmation requires MS/MS fragmentation and/or authentic standards.

[α]Total sum of peak abundance and % relative abundance of these compounds in the negative ESI mode.
