# Supplementary material for: Phytochemical Profile and Antioxidant Potential of Montanoa bipinnatifida C. Koch Leaf Extract: Promising Bioactives for Pharmaceutical Applications
Source: Antioxidants (Basel). 2026 May 8;15(5):598. doi: 10.3390/antiox15050598 (PMC13203521; doi:10.3390/antiox15050598)
Supplement: Supplementary file 1 [file antioxidants-15-00598-s001.zip › Suplemmentary material (S2) GC-MS.pdf]

SUPLEMMENTARY MATERIAL (S2)

Table S4. Volatile compounds identified in MB-LE.

Name of the compound	MW (g/mol)	Molecular formula	Rt (min)	Peak area %
Caryophyllene	204.35	$C_{15}H_{24}$	19.82	0.31
Caryophyllene oxide	220.35	$C_{15}H_{24}O$	22.47	0.80
Lauroyl peroxide	398.62	$C_{22}H_{46}C_2O_6$	23.57	0.81
1-octadecyne	250.50	$C_{18}H_{34}$	26.00	1.15
7-octadecyne, 2-methyl-	266.50	$C_{19}H_{38}$	26.33	0.74
3,7,11,15-tetramethyl-2-hexadecen-1-ol	296.53	$C_{20}H_{40}O$	26.55	0.44
N-hexadecanoic acid	256.42	$C_{16}H_{32}O_2$	27.63	1.51
Methyl 2,8-dimethyltridecanoate	228.37	$C_{14}H_{28}O_2$	27.97	9.42
A'-neogammacer-22(29)-ene	410.73	$C_{30}H_{50}$	28.21	36.22
Bicyclo[4.4.0] dec-5-ene,1,5-dimethyl-3-hydroxy-8-(1- methylene-2-hydroxyethyl-1)-	236.36	$C_{15}H_{24}O_2$	28.67	1.58
Alloaromadendrene oxide-(2)	220.35	$C_{15}H_{24}O$	29.2	0.80
Phytol	296.53	$C_{20}H_{40}O$	29.43	3.65
5,8,11,14,17-ecosapentaenoic acid	302.45	$C_{20}H_{30}O_2$	29.84	13.67
5-chlorovaleric acid, dodec-9-ynyl ester	258.78	$C_{14}H_{23}ClO_2$	29.96	9.65
1-naphthalenepropanol, alpha.-ethyldecahydro-5- (hydroxymethyl)-alpha.,5,8a-trimethy	290.48	$C_{20}H_{34}O$	30.04	11.36
Octadecanoic acid, ethyl ester	312.53	$C_{20}H_{40}O_2$	30.33	1.08
Hexadecane, 1-chloro	260.89	$C_{16}H_{33}Cl$	32.32	0.22
Docosa-8,14-diyn-cis-1,22-diol, bis(trimethylsilyl) ether	478.9	$C_{28}H_{54}O_2Si_2$	32.89	1.89
Eicosanoic acid, 2,3-bis [(trimethylsilyl)oxy] propyl ester	530.97	$C_{29}H_{62}O_4Si_2$	33.97	0.46
Card-20(22)-enolide, 3,5,14,19-tetrahydroxy-, (3-beta,5-beta)-	406.52	$C_{23}H_{34}O_6$	34.22	3.92
Cholest-2-ene	370.70	$C_{27}H_{46}$	34.67	0.33
			**TOTAL**	**100**

Table S5 Non-volatile compounds identified from the derivatized MB-LE.

Name of the compound	MW (g/mol)	Molecular formula	Rt (min)	Peak area %
Silane	32.12	SiH4	4.49	0.45
Trifluoroacetamide	113.04	$C_2H_2F_3NO$	5.09	0.12
Acetamide, 2,2,2-trifluoro-n	113.04	$C_2H_2F_3NO$	5.22	0.26
Ethane	30.07	C_2H_6	5.66	0.72
Propanoic acid	74.08	$C_3H_6O_2$	7.23	1.51
Acetic acid	60.052	$C_2H_4O_2$	7.54	0.39
Benzoic acid	112.12	$C_7H_6O_2$	9.52	0.50
Glycine	75.07	$C_2H_5NO_2$	10.06	1.31
Glycerol	92.09	$C_3H_8O_3$	11.4	7.12
D-Threitol	122.12	$C_4H_{10}O_4$	15.42	1.69
Ribitol	152.14	$C_5H_{12}O_5$	18.43	0.76
Arabitol	152.14	$C_5H_{12}O_5$	18.6	8.56
Beta-dl-arabinopyranose	150.13	$C_5H_{10}O_5$	19.76	1.56
Isocitric acid lactone	174.11	$C_6H_6O_6$	20.48	0.40
D-galactose	180.16	$C_6H_{12}O_6$	20.87	0.91
D-mannopyranose	180.16	$C_6H_{12}O_6$	21.04	0.29
D-mannitol	182.17	$C_6H_{14}O_6$	21.4	3.26
Glucitol	182.17	$C_6H_{14}O_6$	21.49	0.57
Hexadecanoic acid	256.43	$C_{16}H_{32}O_2$	22.42	2.99
Androst-5-ene	258.40	$C_{19}H_{30}$	23.2	8.34
13-cis-retinoic acid	286.50	$C_{20}H_{30}O$	23.74	18.53
Bicyclo[4.4.0]dec-6-en-9beta-ol, 1,7-dimetil-4alfa-isopropenyl	262.40	$C_{17}H_{26}O_2$	24.11	1.43
10,12-docosadiynedioic acid	362.50	$C_{22}H_{34}O_4$	24.34	2.00
Octadecanoic acid	284.50	$C_{18}H_{36}O_2$	24.69	1.81
Cis-5,8,11,14,17-eicosapentaenoic acid	302.50	$C_{20}H_{30}O_2$	26.03	2.03
1,3-dipalmitin	568.90	$C_{35}H_{68}O_5$	28.39	3.10
Allonic acid lactone	178.14	$C_6H_{10}O_6$	29.32	0.32
1-monooleoylglycerol	356.50	$C_{21}H_{40}O_4$	29.98	0.74
D-turanose	342.30	$C_{12}H_{22}O_{11}$	30.29	15.77
Pregnane	288.50	$C_{21}H_{36}$	30.85	1.10
Docosanoic acid	340.60	$C_{22}H_{44}O_2$	32.88	1.13
Betulin	442.70	$C_{30}H_{50}O_2$	33.34	5.27
Stigmasterol	412.70	$C_{29}H_{48}O$	34.73	5.05
			**TOTAL**	**100**
